# Molecular Processes That Control Organic Electrosynthesis in Near-Electrode Microenvironments

**DOI:** 10.1021/jacs.4c14420

**Published:** 2025-01-27

**Authors:** Ricardo Mathison, Rasha Atwi, Hannah B. McConnell, Emilio Ochoa, Elina Rani, Toshihiro Akashige, Jason A. Röhr, André D. Taylor, Claudia E. Avalos, Eray S. Aydil, Nav Nidhi Rajput, Miguel A. Modestino

**Affiliations:** † Department of Chemical and Biomolecular Engineering, New York University, Brooklyn, New York 11201, United States; ‡ Department of Materials Science and Chemical Engineering, Stony Brook University, Stony Brook, New York 11794, United States; § General Engineering, New York University, Brooklyn, New York 11201, United States; ∥ Department of Chemistry, New York University, New York, New York 10003, United States

## Abstract

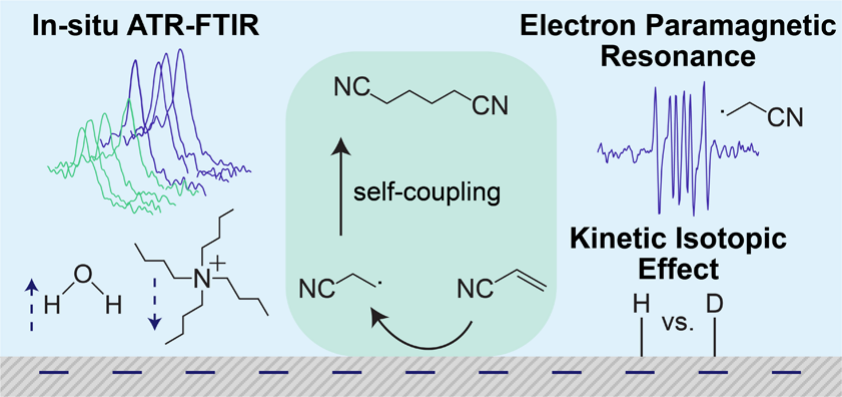

Electrosynthesis at an industrial scale offers an opportunity to use renewable electricity in chemical manufacturing, accelerating the decarbonization of large-scale chemical processes. Organic electrosynthesis can improve product selectivity, reduce reaction steps, and minimize waste byproducts. Electrochemical synthesis of adiponitrile (ADN) via hydrodimerization of acrylonitrile (AN) is a prominent example of industrial organic electrochemical processes, with annual production reaching 0.3 MMT. It circumvents the drawbacks of thermochemical synthesis by reducing toxicity and leveraging clean electricity as an energy source. Despite its industrial importance, mechanistic understanding and experimental insights on the near-electrode molecular processes of AN electrohydrodimerization remain insufficient. Here we show, using in situ ATR-FTIR spectroscopy, that tetraalkylammonium ions populate the electrical double layer (EDL), creating a microenvironment that favors interactions with organic molecules and enhances AN concentration while expelling water molecules. Our results provide experimental evidence supporting long-standing mechanistic hypotheses. Kinetic isotope effect studies reveal that propionitrile (PN) formation is rate-limited by proton transfer, while ADN formation likely is not. Electron paramagnetic resonance spectroscopy confirms the presence of free radicals during AN electroreduction, suggesting that coupling of PN radicals occurs primarily in the electrolyte. These insights highlight the importance of carefully controlling the EDL composition for selective organic electrosynthesis and provide fundamental engineering guidance for designing high-performing electro-organic reactions. We anticipate these findings will guide the optimization of electrolyte formulations and electrode interfaces for ADN synthesis and other emerging electro-organic processes.

## Introduction

Introducing electrosynthesis at an industrial scale represents an opportunity for using renewable electricity directly in chemical manufacturing plants, accelerating the decarbonization of large-scale chemical processes.^1−7^ The greatest potential for decarbonization lies in expanding the electrochemical synthesis of organic chemicals from laboratory-scale research to industrial application, which has only been achieved for a few processes.^2,7,8^ Organic electrosynthesis can offer several advantages, including improvements in product selectivity, reduction of reaction steps and energy use, and minimization of waste byproducts.^8,9^ Electrochemical synthesis of adiponitrile (ADN) via hydrodimerization of acrylonitrile (AN) is a prominent example of industrial organic electrochemical processes.^10^ ADN is a key intermediate in the production of Nylon-6,6, a polymer essential for high-performance textiles and structural materials,^11^ which is primarily produced via the thermochemical hydrocyanation of 1,3 butadiene, a process that is energy intensive and requires highly toxic reactants.^12−14^ The electrohydrodimerization of AN was discovered during the 1950s and 1960s^15−17^ and was later developed and implemented at an industrial scale by the Monsanto Company. Over the past two decades, this process has reached a global production of 300,000 tons per year.^18,19^ Electrochemical synthesis of ADN circumvents the drawbacks of thermochemical synthesis by reducing toxicity and leverages clean electricity as an energy source, thus making it the most successful organic electrosynthesis process commercialized.^8,19^ However, despite the industrial importance of the electrohydrodimerization of AN, our current mechanistic understanding and experimental insights into the molecular processes occurring near the electrode during this reaction remain limited, preventing us to extend the learnings from this successful electrochemical process to the manufacture of other organic chemical products.

**Scheme 1:**
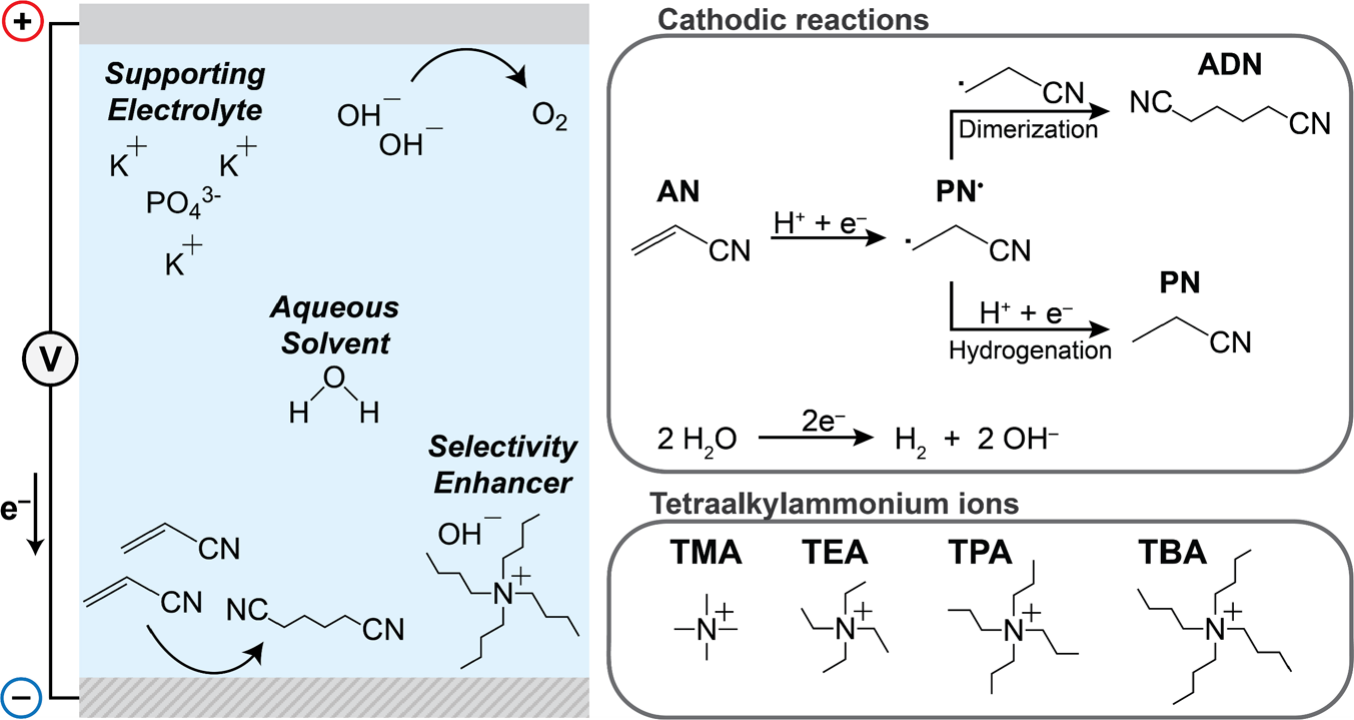
Electrochemical Hydrodimerization of AN Cell scheme of the electrosynthesis of ADN from AN, showing the main electrochemical reactions and electrolyte components. Proposed mechanism of the cathodic reactions present in AN electrohydrodimerization.

Electrohydrodimerization of AN is typically carried out in aqueous solvents and begins with the reduction and protonation of AN’s carbon–carbon double bond to form a radical, followed by their dimerization to produce ADN (Scheme 1). Instead of dimerization, the intermediate radical can also undergo further reduction and protonation, leading to the hydrogenation product, propionitrile (PN), the most common organic side product of the process. In addition, AN-derived oligomers are formed at low current densities and high local concentrations of AN. The hydrogen evolution reaction (HER) is the parasitic solvent reaction at the cathode surface. The pathway toward ADN is promoted under moderate concentrations of AN in the electrical double layer (EDL), which leads to larger rates of radical coupling, effectively preventing further reduction to produce PN and avoiding reactant accumulation that enhances oligomer formation. The first demonstrations of the reaction had discouragingly low yields until Baizer and co-workers enhanced the performance of the process by using quaternary ammonium salts as the supporting electrolyte.^16,20,21^ These salts significantly improved the solubility of AN in aqueous solvent, resulting in nearly quantitative yields and current efficiencies >90%, which led to the successful commercialization of the process.^20,21^ These organic cations are believed to control the environment in the EDL by expelling water, suppressing the HER, and thus promoting the formation of PN radicals.

Modern production of ADN uses electrolytes combining phosphate buffers for pH control and improved conductivity, tetraalkylammonium (TAA) salts for selectivity enhancement and cathode corrosion prevention,^18,22^ and chelating agents to prevent undesired metal cation electrodeposition^23^ (Scheme 1). Cadmium or lead cathodes are preferred for their high overpotential for hydrogen evolution, minimizing this side reaction.^2,21,24,25^ Recent bulk electrolysis investigations have focused on enhancing and understanding the performance of the reaction to improve the competitiveness against the current leading thermochemical process. These investigations provided a better understanding of the effects of electrolyte components, organic spectator cations,^26^ and inorganic supporting cations^27^ to control the concentration of reactive species at the electrode interface to enhance the selectivity toward ADN production. Additionally, strategies to mitigate mass transport limitations have been explored using dynamic operation with potential pulses, informed by machine learning,^28^ and improved convection methods for selectivity control.^29,30^ Despite these advances, our fundamental understanding of reaction mechanisms at the molecular scale remains limited, particularly regarding the role of the substrate and spectator ions in the EDL. Recent efforts aimed at bridging this knowledge gap include computational studies that implement Density Functional Theory (DFT) and Molecular Dynamics (MD) to explore reaction mechanisms on lead surfaces^31−33^ and continuum modeling of operating parameters’ effects on product selectivity.^34^ While bulk studies and computational approaches have advanced our knowledge of molecular interactions and selectivity, we still lack experimental evidence on how electrochemical conditions control species concentration in the near-electrode region and the dominant rate-limiting steps in this reaction.

In this study, we leveraged reaction engineering approaches and in situ spectroscopy tools to investigate the complex phenomena occurring at the electrode surface during AN electrohydrodimerization. Using an electrochemical reactor integrated with Attenuated Total Reflectance Fourier Transform Infrared Spectroscopy (ATR-FTIR), we explored changes in local concentrations of reactants, supporting electrolytes, and the solvent during the reaction, which together with MD simulations allowed us to derive insights into the role of electrolyte species in controlling electrode–electrolyte microenvironments. Additionally, kinetic isotopic effects (KIE) and isotopic labeling studies were employed to understand the role of the protonation steps within the reaction mechanism. Lastly, we used Electron Paramagnetic Resonance (EPR) spectroscopy to determine the role of free radicals during electrolysis. These experimental studies significantly enhanced our understanding of near-electrode microenvironments in AN electrohydrodimerization, offering insights broadly applicable to the electrochemical reduction of activated olefins and other organic reductions in aqueous media.

## Results and Discussion

### Elucidating Near-Electrode Microenvironments via In Situ ATR-FTIR

**Figure 1:**
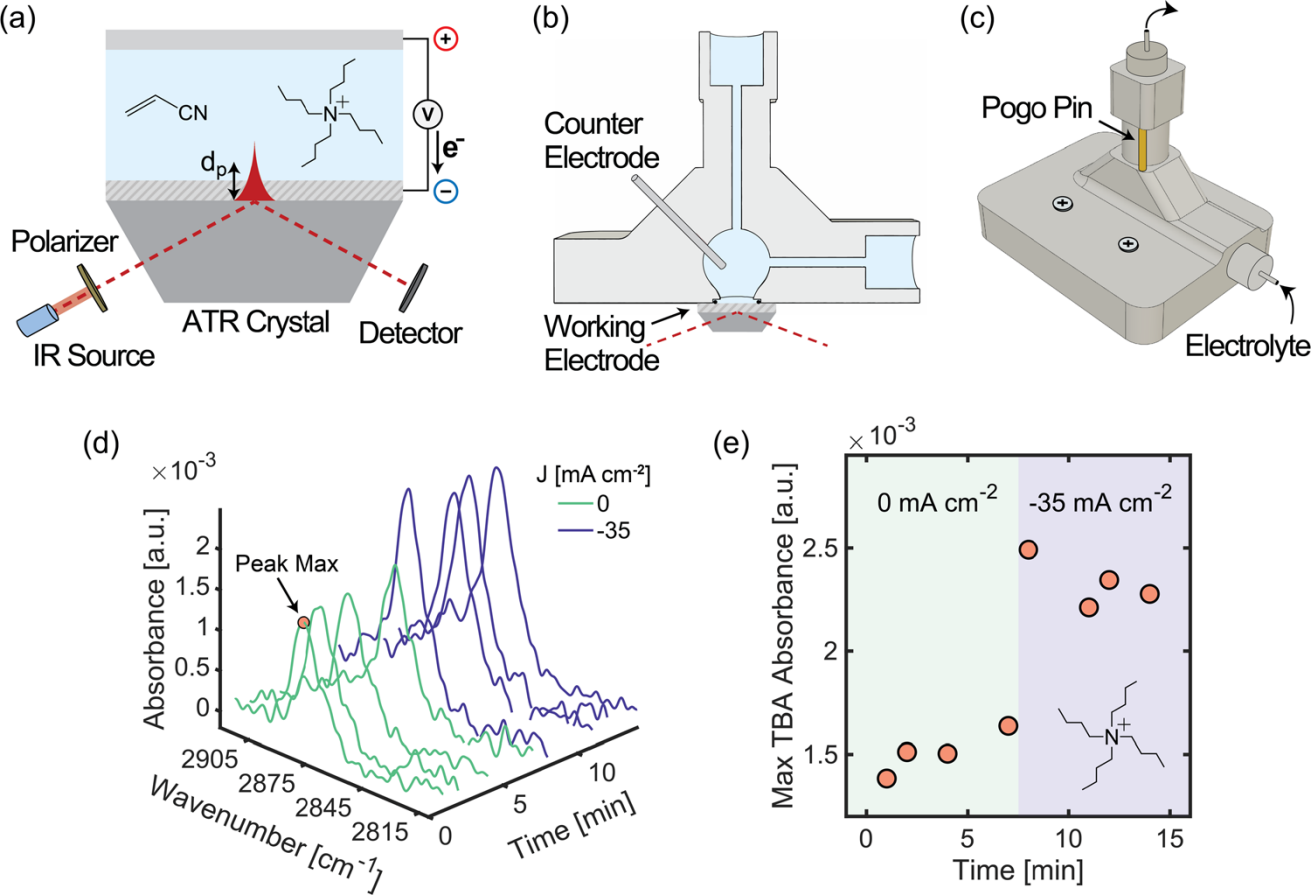
Local concentration assessment via ATR-FTIR. (a) Schematic representation of the electrochemical ATR-FTIR cell. (b, c) Cross-sectional and isometric view of the integrated ATR-FTIR electrochemical reactor used in this study showing the main design components, such as the reaction chamber, the location of the working electrode and counter electrode, the direction of flow, and the location of the pogo pins used to make contact with the cathode. (d) Time evolution of the recorded IR spectra of near-electrode TBA ions under current densities of either 0 or −35 mA cm^–2^ highlighting the maximum absorbance of the symmetric CH_3_ stretch that is used for data visualization in this study. The electrolyte contained 0.05 M TBA hydroxide. (e) Time evolution of the maximum absorbance of the symmetric CH_3_ stretch in TBA.

The concentration of reactive species in the EDL and electrode potentials determine the reactive pathways in organic electrosynthesis. In the context of the electroreduction of AN, the key is to enhance the presence of AN in the near-electrode region to promote dimerization, thus favoring the formation and coupling of PN radicals and suppressing full hydrogenation of the olefin and HER. Decades of research led to empirical electrolyte design rules that favor those conditions. Informed by the empirically optimized conditions and strategies, we investigate the fundamental origins for the success of these conditions to translate the learnings broadly into other organic electrosynthesis. To that end, we study and establish relationships between electrolyte composition, current density, and species concentration in the near-electrode region by locally probing the electrode/electrolyte interface. Prior studies successfully implemented spectroelectrochemical approaches to elucidate unknown concentrations of intermediates, products, and supporting molecules at electrochemical interfaces.^35−37^ Inspired by these studies, we designed and fabricated an electrochemical flow cell integrated with an ATR-FTIR spectrometer to characterize species in the near-electrode region. This spectroelectrochemical cell consisted of a single reflection ATR Si crystal^38^ as the internal reflection element integrated with a flow electrochemical cell fabricated in-house (Figure 1a–c). A single reflection crystal was used, given the need for a metallic surface and a short, effective path length in the presence of water, which is a strong IR absorber.^39^ We were not able to achieve appropriate light penetration and strong substrate addition through Cd or Pb electrodes, leading to the selection of a thin two-layer metal film (5 nm Cr followed by 7 nm Ag) that was deposited onto the ATR Si crystal through thermal evaporation and served as the working electrode for the cell. The electrode was thick enough to operate at current densities of tens of mA cm^–2^ without delaminating from the crystal and thin enough to let the evanescent wave penetrate the metal and interact with molecules at and near the electrode surface. This configuration allowed us to track changes in the local concentration of TAA ions with bulk concentrations as low as 0.01 M. In this configuration, the penetration depth, *d*_p_, was at most 1100 and 300 nm at 1000 and 4000 cm^–1^, respectively, which is expected to be lower due to the light absorbed by the electrode (observed by a decrease in intensity of the background before and after deposition). In comparison, for a 0.05 M TBA electrolyte solution, the EDL thickness is 1.4 nm and the OHP is 0.5 nm from the electrode surface.

With this setup, we could track changes in local concentrations of reactants and spectator ions near the electrode surface. Accurate electrode potential measurements in our spectroelectrochemical cell were hindered by high resistance (100–1000s ohm) stemming from thin electrodes, organic electrolyte resistivity, and space constraints. Due to these limitations, we focused our analysis on current densities, which could be measured with high precision. While surface adsorbed species and activity of this Cr/Ag electrode are different from that of Cd or Pb electrodes used in industrial processes, the electric field impact on the structure and composition of the near electrode region is expected to be similar. This is supported by Wu and co-workers’^31^ demonstration that bulk AN concentration critically determines product distribution across multiple electrode materials (Pb, Ag, Cu), with significant changes observed between 0.05 and 0.5 M AN regardless of the cathode material used, and the suppression of ADN production in the lower concentration range. These results suggest that the microenvironment’s influence on reactivity transcends surface-specific effects. In this study, we selected operating conditions based on our previous systematic studies of bulk reaction parameters.^26,27,30^ To complement our spectroscopic findings, we have included bulk electrolysis product distributions in Figure S1, varying key parameters including TAA ion chain length, cathode material, current density, and AN concentration. While these works established optimal conditions for ADN formation, the molecular-level behavior at the electrode surface remained unclear. Our focus here is to understand how reactive species and spectator ions organize in the electrode microenvironment under these relevant conditions, providing insights into the local environment that leads to the observed product distributions.

All in situ results presented in this study were taken at a constant electrolyte flow rate of 0.32 mL s^–1^. For each condition (electrolyte composition and applied current density), multiple ATR-FTIR spectra were taken over minutes to ensure that the concentration of species in the near-electrode surface was approaching steady state (Figure 1d). Spectra of TAA, AN, and phosphate ions are represented as differences with respect to a DI water background spectrum, while water spectra are shown as differences from an air background spectrum, taken less than 2 min before each experiment set. The maximum of each peak was identified and plotted as a function of time to establish relationships between bulk experimental conditions and changes in local concentrations, given the proportionality defined by Beer’s Law (Figure 1e). For all experiments conducted in the absence of AN, HER is expected to be the only electron transfer process occurring at the electrode surface. At cathodic current densities greater than 1 mA cm^–2^ there was a shift in the baseline of the spectra due to polarization effects on thin metal films, which can influence the interaction between incident IR radiation and surface electrons,^40−42^ that motivated us to develop a baseline-correction algorithm that deconvolutes peaks from the background allowing comparison of spectra taken at different current densities (details are in Figures S2 and S3). Full-range FTIR spectra (800–4000 cm^–1^) are provided in Figures S4–S6, offering a comprehensive view of all spectroscopic changes.

The addition of TAA ions in the electrolyte enhances the solubility of organic reactants and promotes high selectivity toward organic products by controlling the environment near the EDL.^2,26^ These ions display high solubility in aqueous solvent, are electrochemically inert, and can be systematically controlled in size due to their tunable hydrophobic alkyl chains.^43^ The effects of TAA ions on electrochemical interfaces have also been studied in recent years in the context of CO and CO_2_ electroreduction.^44−47^ Cations are expected to populate the outer Helmholtz plane (OHP) of the EDL at cathodic potentials due to the long-range electrostatic attraction^48−50^ toward the electrode and shorter-range van der Waals interactions between organic cations and the electrode surface driving their adsorption.^51,52^ Experimental studies have shown that electrostatically bound TAA ions can interact and displace adsorbed species from surface sites.^44,52,53^ Leveraging our spectroelectrochemical tool, we tested the effect of a polarized cathode on the near-electrode concentration of TAA ions using tetrabutylammonium (TBA) as an example. The local concentration of TBA significantly increased after a current density of −35 mA cm^–2^ was applied at a TBA bulk concentration of 0.05 M (Figure 1e). The negatively charged electrode attracted positive ions, which migrated toward the surface electrode and populated the OHP and diffusive layers. The presence of TBA in the EDL is expected to create an organic-rich microenvironment that will boost the local concentration of AN, thus favoring electron transfer reactions with organic reactants over water.

### Effect of Current Density on the Near-Electrode TBA Concentration

**Figure 2:**
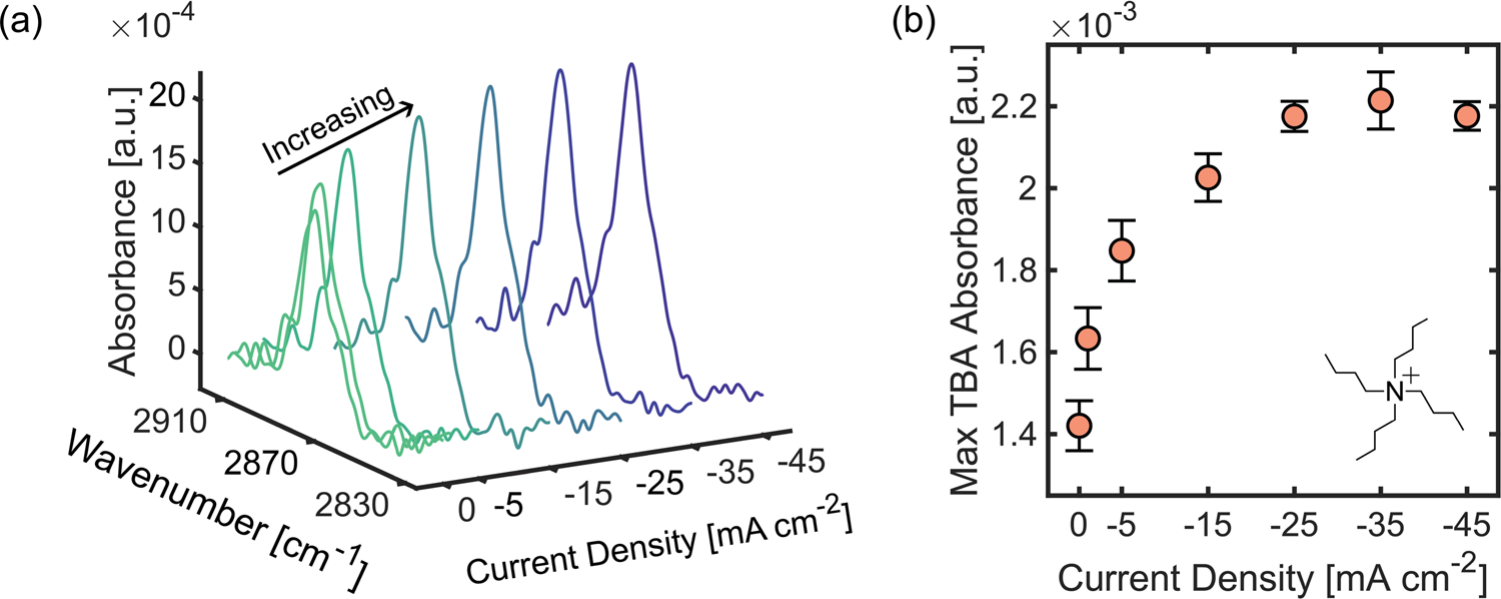
Effect of current density on TBA near-electrode concentration. (a) IR spectra of near-electrode TBA ions as a function of applied cathodic currents from 0 to −45 mA cm^–2^. Multiple spectra were obtained for each experimental condition to confirm that the local concentration approached steady-state. The electrolyte contained 0.05 M TBA hydroxide. (b) Effect of applied cathodic current on the near-electrode concentration of TBA ions depicted by the maximum absorbance of the symmetric CH_3_ stretch.

Having demonstrated that the application of cathodic potentials increased the local concentration of TBA in the near-electrode surface, we explored how varying current density from 0 to −45 mA cm^–2^ changed the local TBA concentration quantitatively (Figure 2). When no potential is applied, the near-electrode concentration of TBA is expected to approach the bulk concentration so that the absorbance value measured corresponds to the bulk molarity. The TBA absorbance increases steeply with increasing current density as TBA ions migrate to the electrode surface (between −1 and −10 mA cm^–2^) but eventually saturates at moderate current densities (between −20 and −45 mA cm^–2^). Since the distance above the electrode surface probed by ATR-FTIR goes beyond the EDL, and the bulk concentration is expected to remain constant, we conclude that the local concentration of TBA in OHP increases and then saturates as the current increases. We attribute the saturation to the electrostatic repulsion and steric hindrance between TBA ions, which makes their adsorption strongly coverage dependent,^52^ becoming less favorable with increasing coverage and more favorable at lower electrode potentials due to reduced crowding of the OHP by organic cations.^48^ The TBA absorption maxima in chronological order of experiments where the spectra were collected for ascending and descending current densities are shown in Figure S7. The effect of the cathodic current on the local TBA concentration is independent of the direction in which the potential is swept, confirming that each measurement is independent of previous experimental conditions. In both cases, the reactor was brought back to an open circuit condition after the sweep was over (0 mA cm^–2^), and while the local concentration of TBA decreased, it did not return to the original concentration before the sweep was conducted. This behavior shows the strong effect of the polarized electrode on the diffusion of TBA away from the EDL. Figure S8 shows the same trend on the effect of cathodic potential on near-electrode TBA concentration for different bulk concentrations. The changes observed in the local TBA concentration near the electrode are consistent with electrochemical impedance spectroscopy (EIS) studies, which show changes in the double-layer capacitance under cathodic conditions, attributed to the adsorption of TBA ions, leading to the displacement of hydronium ions and thus increasing local pH.^46,54,55^ These results confirm the enhanced presence of TAA ions in the negatively charged electrode interface due to electrostatic attraction.

### Effect of TAA Ions on Near-Electrode AN Concentration

ADN electrosynthesis becomes mass transport limited at moderate current densities because of the low solubility of AN in water, and AN dimerization requires two equivalents of the substrate. It is accepted that TAA ions enhance the solubility of AN in the aqueous phase, thus leading to a higher concentration near the electrode surface. These organic cations with hydrophobic chains are known to create a hydrophobic layer at the interface,^45^ which is expected to enhance the concentration of organic reactants due to favorable van der Waals interactions^27,28^ and repel water from this region. While large concentrations of TAA ions can help enhance the solubility of AN, at high concentrations, TAA ions can decompose at the anode, thus making the process economically unfeasible.^22^ However, small concentrations are sufficient to promote the selectivity toward ADN significantly. Recent experiments show that ADN production rates are negligible without TAA ions, but adding TAA at concentrations as low as 5 mM can result in ADN being the most favored product.^27,31^

**Figure 3:**
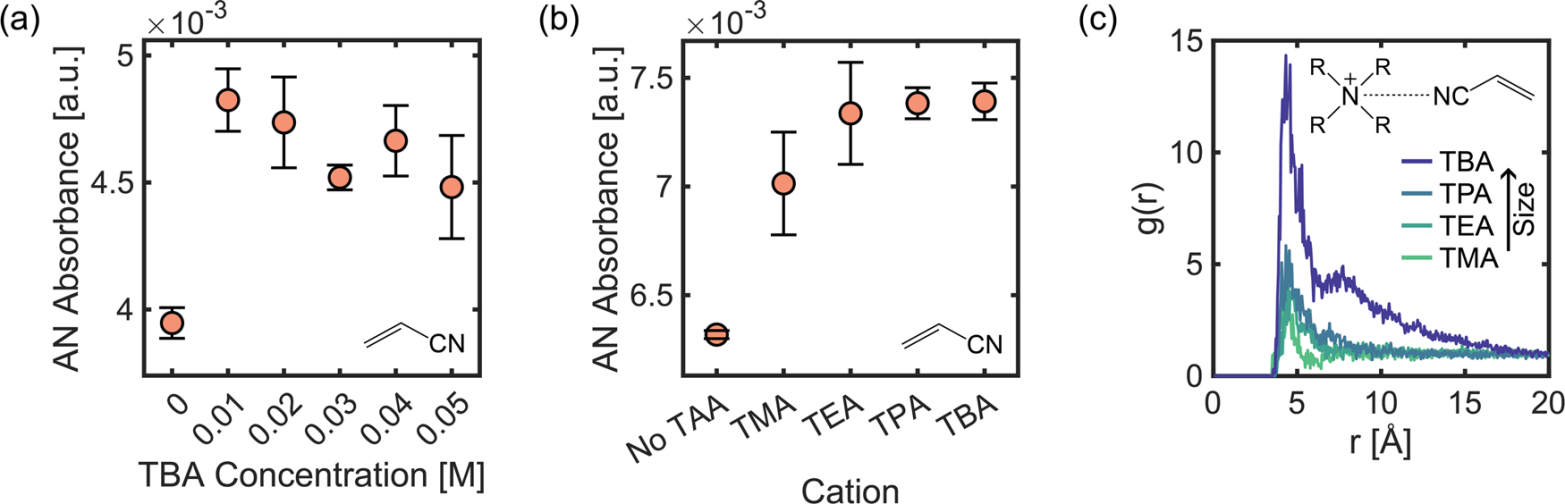
Effect of TAA ions on AN near-electrode concentration. (a) Effect of TBA ion bulk concentration on the near-electrode concentration of AN depicted by the maximum absorbance of the C≡N stretch. The electrolyte contained 0.6 M AN, 0.5 M K_3_PO_4_, and the TBA hydroxide concentration shown above. (b) Effect of TAA ion size on the near-electrode concentration of AN depicted by the maximum absorbance of the C≡N stretch. The electrolyte contained 0.6 M AN, 0.04 M TAA hydroxide (except for No TAA), and 0.5 M K_3_PO_4_. For both experiments, multiple spectra were obtained under a current density of −25 mA cm^–2^ for each experimental condition to confirm that the local concentration approached steady-state. (c) Radial distribution functions of N (TAA)–N (AN) obtained from MD simulations of electrolytes composed of 0.6 M AN, 0.5 M K_3_PO_4_, and 0.05 M TAA–OH in water.

We tested the effect of TBA ions on the local concentration of AN during electrolysis while conducting chronopotentiometry (CP) experiments at −25 mA cm^–2^, as TBA concentrations were varied from 0 to 0.05 M while all other experimental variables were kept constant (Figure 3a). The near-electrode AN concentration showed a significant increase with as little as 0.01 M TBA added to the electrolyte, remaining around the same value in the 0.01–0.05 M TBA concentration range. When there are no TBA ions present in the electrolyte, the local concentration of AN is lower and as a result there is no production of ADN in the bulk as seen in Figure S1. Under the same conditions, addition of 0.02 M of TBA ions in the electrolyte leads to ADN being the favored product. This is consistent with reported product distributions, where small amounts of TBA ions drastically enhanced selectivity toward ADN, but the concentration dependence with subsequent increases was weak.^31^Figures S9 and S10 show the spectra and data in chronological order of the experiments used to construct Figure 3.

We studied the effect of TAA ion size on the local concentration of AN by changing the TAA ion’s alkyl chain length from “zero” (no TAA ion present in the electrolyte) to four carbons (TBA) and performed CP experiments at −25 mA cm^–2^. The local concentration of AN increased with alkyl chain length (Figure 3b). This is consistent with an increasingly nonpolar environment with longer hydrophobic chains in the OHP that enhances the AN solubility due to stronger van der Waals interactions. Similar to the observations varying TBA concentration, the largest local AN concentration difference is observed between the electrolytes with and without TAA ions present. Addition of TMA ions to the electrolyte lead to an increase in the local concentration of AN, though not substantially enough promote the dimerization rate toward ADN (Figure S1). This trend is consistent with observations from bulk electrolysis experiments where reaction rates toward organic products increased with TAA ion size.^26^

The increased affinity of AN with TAA ions of larger size was confirmed by MD simulations. Radial distribution functions, *g*(*r*) shown in Figure 3c, around nitrogen atoms of AN and TAA ions were computed to help interpret the observed experimental results. The strength of interaction between AN and TAA ions increases with alkyl chain length, leading to the observed behavior where larger TAA ions enhance the local concentration of AN in the electrode–electrolyte interface.

### Effect of Electrolyte Cations on Near-Electrode Water Concentration

ADN is typically electro-synthesized in water-based electrolytes, allowing large concentrations of supporting ions, lower ionic resistance, and impro ved processing economics and sustainability. Water is the proton source for forming organic products (ADN, PN, and oligomers) and HER. Selectivity toward ADN tends to be compromised by competition with the HER. Strategies to suppress HER include electrode selection and electrolyte design. Higher charge density cations bind water more tightly in their solvation shells, affecting surface charge density at electrodes and HER electrocatalytic activity. In addition, the stability of intermediate radicals and anions in cathodic electro-organic reactions is influenced by solvating cations near the electrode.^27^

Alkali cations have been shown to have a large effect on product distributions in organic electro-reductions, which has inspired recent studies. The size of alkali cations significantly affects faradaic efficiencies, mostly due to variations in the potential of the OHP and steric hindrance effects.^56^ DFT calculations indicate that larger hydrated cations are more energetically favored at the OHP, suggesting a larger coverage of cations as cation size increases.^57^ The EDL chemical composition is further controlled by the addition of TAA ions, which, under cathodic conditions, are known to populate the OHP, as demonstrated in the results described above. The hydrophobic and steric effects from quaternary ammonium salts displace interfacial water molecules and effectively suppress HER,^46,58^ with increasing chain lengths leading to greater HER suppression.^59^ This is due to the ability of TAA ions to block electrode surface sites, hindering the adsorption of hydrogen and water.^52,60^

**Figure 4:**
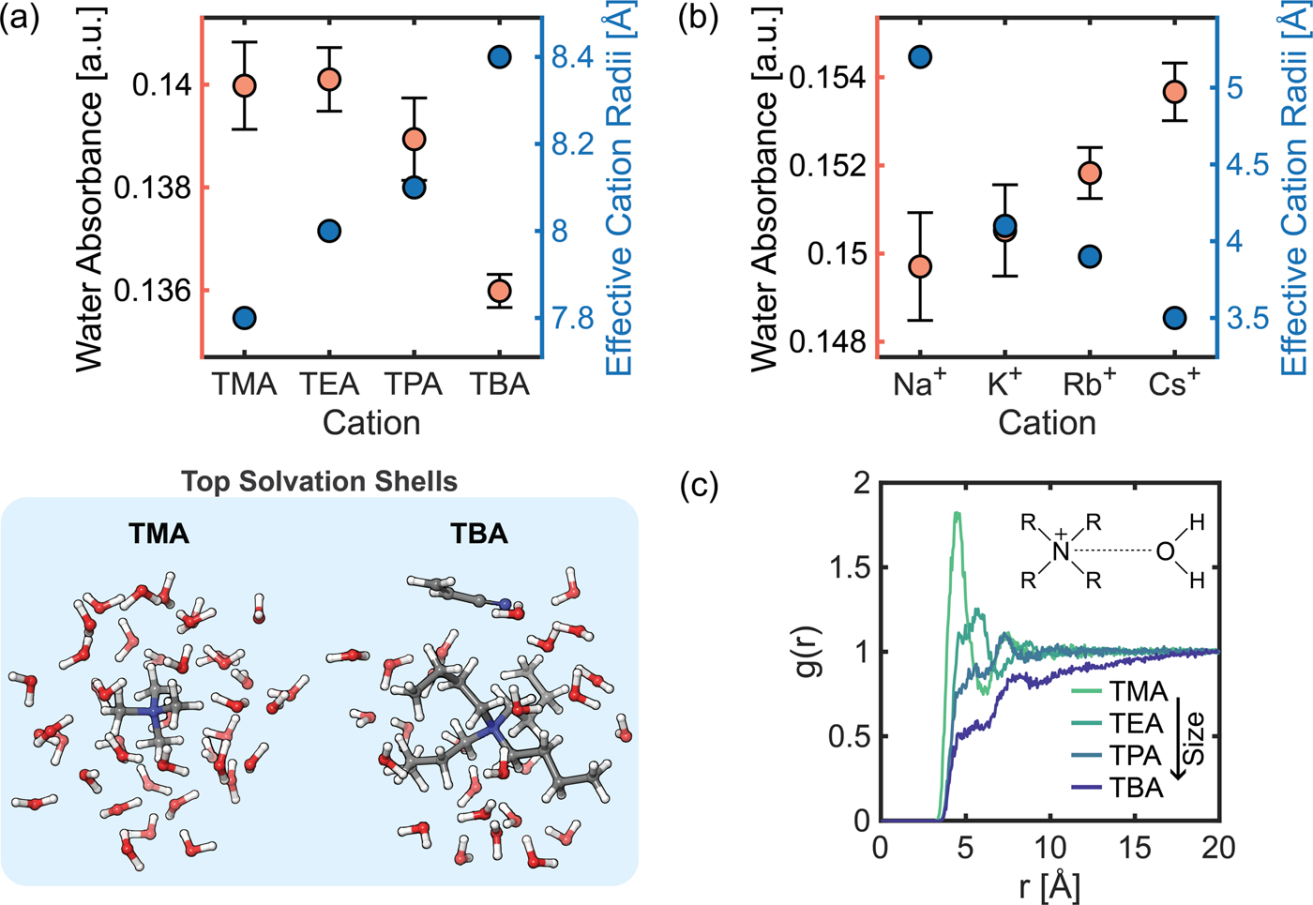
Effect of electrolyte cations on water near-electrode concentration. (a) Effect of TAA ion size on the local concentration of water under a cathodic current. The electrolyte contained 0.05 M TAA hydroxide. (b) Effect of alkali cation size on the local concentration of water under a cathodic current. The electrolyte contained 0.5 M (Cation)_3_PO_4_. For all experiments, the current density was −25 mA cm^–2^. The local concentration of water is depicted by the maximum absorbance of the O–H scissor, and multiple spectra were obtained for each experimental condition to confirm that the local concentration approached steady-state. Effective interfacial cation radii were reproduced with permission from ref (56). Copyright 2019 The Royal Society of Chemistry. (c) Radial distribution functions of N (TAA)–O (H_2_O) obtained from MD simulations of electrolytes composed of 0.6 M AN, 0.5 M K_3_PO_4_, and 0.05 M TAA–OH in water.

In the context of ADN electrosynthesis, the aqueous electrolyte contains a combination of alkali and TAA ions, and their size is known to have a strong effect on product distributions. We tested the effect of both inorganic and organic cations on the local water concentration, which is expected to affect HER rates. The TAA ions’ alkyl chain length was varied from one (TMA) to four carbons (TBA) while we performed CP experiments at −25 mA cm^–2^. The near-electrode concentration of water decreased with increasing alkyl chain length, with the more hydrophobic ions repelling water molecules more effectively (Figure 4a). The trend of local water concentration has a close agreement with the effective interfacial cation radii,^56,61−63^ showing that larger TAA ions exclude water from the EDL and in turn promote organic reactions. We performed an analogous experiment increasing the size of alkali cations (Na^+^, K^+^, Rb^+^, and Cs^+^) under cathodic conditions. The local concentration of water increased with larger alkali cations but had an opposite trend with respect to the effective cation radii (Figure 4b). Hydrated alkali cations of larger size have been shown to form a more compact layer at the electrode–electrolyte interface,^56,57^ which in turn controls the availability of water molecules in that region. Our previous study showed that smaller alkali cations enhance the stability of radical AN-derived anions, thus prolonging their lifetime and favoring coupling toward ADN.^27^ Balancing the effects of water availability and organic intermediate stabilization is critical for selective electrosynthesis at high current densities. We employed MD simulations to investigate the interaction between water molecules and TAA ions of varying sizes. Analysis of the radial distribution functions, *g*(*r*), computed around the central nitrogen atoms of TAA ions revealed a size-dependent affinity for water (Figure 4c). The simulations demonstrated that TAA ions with shorter alkyl chains exhibited stronger interactions with water molecules. This computational finding aligns with our experimental results. The enhanced hydration of smaller TAA ions observed in the simulations provides a molecular-level explanation for the relationship between TAA ion size and water content in the electrolyte. The solvation shell of TBA contains both water and an AN molecule, while that of TMA consists solely of water. Additionally, there is a higher degree of overlap in diffusion coefficients of TMA ions with those of water compared to that of alkali cations of longer chain length (Figure S14). The structural and diffusion analysis results indicate that smaller-sized TAA ions can move with their hydration shell to the interface, increasing water availability.

We also explored the effect of current density on local water concentration in the presence of large bulk concentrations of only one cation. Figure S11 shows how, under a sodium phosphate electrolyte, the local concentration of water was observed to drop significantly with a small increment in current density (−1 mA cm^–2^) and continued to decrease asymptotically, an effect that was independent of the size of the cation used (Figure S12). The near-electrode concentration of phosphate ion was tested under similar conditions, with the anions decreasing in local concentration as the cathodic potential increased (Figure S13). This behavior is expected because of the species displacement by cations in the OHP and the repulsion between the anions and the charged cathode. In addition, these combined effects showed reversible behavior. After the reactor was returned to the open circuit, the local concentration of phosphate ions also returned to the original value.

### Exploring Rate-Limiting Steps via Kinetic Isotope Effect Studies

We explored the reaction mechanism and the rate-limiting steps using kinetic isotope effect studies. Multiple studies on the mechanism of AN electroreduction concluded that the dimerization pathway starts with forming a radical anion,^64−66^ a step identified as rate-limiting by a recent kinetic study by Huang and co-workers.^32^ Further experimental and theoretical calculations revealed that the product distribution heavily depends on the competitive adsorption between hydrogen and AN, with ADN being favored when the cathode surface is covered by AN.^31^ Despite significant progress in understanding the reaction’s mechanism, to the best of our knowledge, there is no experimental evidence on the role of proton transfer in AN electroreduction.

To investigate the role of the protonation of AN’s vinyl group in the reaction mechanism, we performed H/D KIE and isotopic incorporation studies. KIE studies monitor how reaction rates are affected when an atom in the reactants is replaced with its isotope.^67^ Isotope effects stem from the disparity in zero-point energies between unlabeled (i.e., C–H and O–H) and labeled (i.e., C–D and O–D) bonds; where the increased mass of a C–D and O–D bonds significantly affect their stretching frequencies, making them lower compared to C–H and O–H bonds and affecting the kinetics of reaction steps involving the formation or cleavage of these bonds.^68^

**Figure 5:**
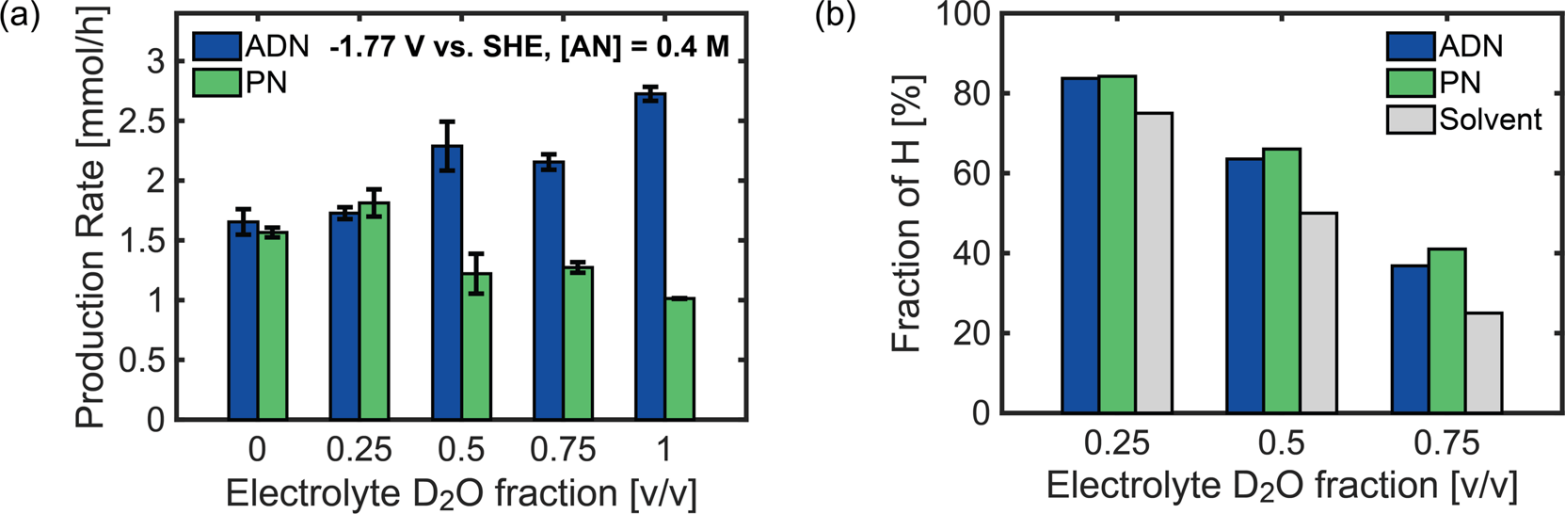
H/D kinetic isotopic effect on acrylonitrile electroreduction. (a) Production rate of adiponitrile (ADN) and propionitrile (PN) for 10 min electrolysis on Cd foil at −1.77 V vs SHE in five electrolytes with varying H/D compositions, depicted by D_2_O volume fraction in the solvent with H_2_O as the remaining solvent. Electrolytes contained 0.4 M AN, 0.5 M Na_3_PO_4_, 0.03 M EDTA, and 0.02 M TBA hydroxide. (b) H/D isotopic compositions in the products after electrolysis and their theoretical values in the electrolyte with different compositions.

We conducted AN electroreduction experiments on Cd foil at −1.77 V_SHE_ (V vs SHE) for 10 min with mixtures of H_2_O and D_2_O, denoted by the fraction of D_2_O that is used as the solvent (where an H_2_O-only electrolyte is represented by a D_2_O fraction of 0). The bulk concentration of AN and electrolyte composition was the same for all experiments. We selected these reaction conditions (−1.77 V vs SHE, −200 mA cm^–2^) as they resulted in similar production rates of ADN and PN in H_2_O solvent, allowing us to track changes in selectivity as the deuterium fraction increased. Linear sweep voltammetry (LSV) profiles of H_2_O vs D_2_O electrolyte (Figure S15) show a decreased reaction rate of deuterium (D_2_) evolution compared to HER, which agrees with previous studies.^69^ The addition of AN to both types of electrolytes significantly decreased the electrode overpotentials, shifting the onset potential (at −1 mA cm^–2^) from −1.57 and −1.43 V_SHE_ for AN-free D_2_O and H_2_O electrolytes, respectively, to −1.28 V_SHE_ for AN-rich electrolytes, regardless of the degree of deuteration of the solvent. Additionally, when AN was present, reaction rates did not significantly change with water deuteration. Isotopic effects were identified with changes in product distributions for AN electroreduction. Figure 5a shows that the production rates of ADN gradually increased from 1.7 to 2.7 mmol h^–1^ with increasing D_2_O fractions, whereas production rates of PN decreased from 1.6 to 1.0 mmol h^–1^. Under the same reaction conditions, product-selective KIE was detected for the formation of both ADN and PN production, indicating that AN electroreduction is rate-limited by proton or hydrogen transfer to reaction intermediates.

We used isotopic labeling to obtain molecular-level information on the selective addition of H/D atoms to AN’s vinyl group. The mass spectra from the gas chromatography–mass spectrometry (GC–MS) analysis are shown in the Supporting Information. Figure S16 shows ADN and PN fragmentation patterns with varying H/D compositions. Ion peaks for both products shifted to higher *m*/*z* values with increasing D_2_O fractions; thus, the solvent used is critical in the isotopic composition of AN-reduction products. We calculated the isotope compositions represented by the fraction of H in the products by quantitatively fitting H and D fractions to GC-MS data, with details in the Supporting Information. The isotopic composition of products under H_2_O/D_2_O electrolyte mixtures is shown in Figure 5b. Both ADN and PN contain consistently higher H-fractions than the electrolyte composition values, indicating that the addition of a proton to AN’s unsaturated bond has higher reaction rates for H than for D. The rate-limiting proton transfer steps could be a result of distinct mechanistic routes that happen simultaneously at varying rates. For example, protonation could occur via proton transfer from a bulk solvent species (i.e., H_2_O or H_3_O^+^) by an intermediate carbanion or by reduction of AN by an adsorbed H preceded by the Volmer step, where water dissociation is coupled with proton adsorption. Both mechanisms require breaking H–O bonds of solvent species. Given that breaking D–O bonds requires more energy,^70−72^ the addition of H over D to the unsaturated bond of AN is preferred, as revealed by the two products analyzed via mass spectrometry.

Compiling the results from KIE and isotope incorporation studies, we have experimentally elucidated the role of protons in the electroreduction of AN. PN formation is rate-limited by a proton transfer, which may come from the surface hydrogenation of AN or the Volmer step, in close agreement with kinetic modeling on Pb electrodes^32^ and consistent with the larger H-fractions on PN compared to ADN. The H/D isotope effect did not influence the rate of electroreduction of AN, as shown by almost identical LSV profiles when using H_2_O and D_2_O-solvent electrolytes and by a relatively constant electrochemical conversion rate of AN across a gradient of D_2_O solvent fractions. These results suggest that proton transfer steps do not limit the AN dimerization, since the second protonation of AN that yields PN could be the rate-limiting step. Our observations provide mechanistic understanding of the selective production of ADN by successfully blocking routes toward PN. Conditions that favor a lower interfacial pH correspond to a higher concentration of proton donors near the electrode,^73^ which enhances HER^74,75^ and the rate of hydrogenation of AN as seen in bulk studies^26,31^ justified by this mechanistic study.

### Understanding the Role of Free Radicals in ADN Electrosynthesis via Electron Paramagnetic Resonance Spectroscopy

**Figure 6:**
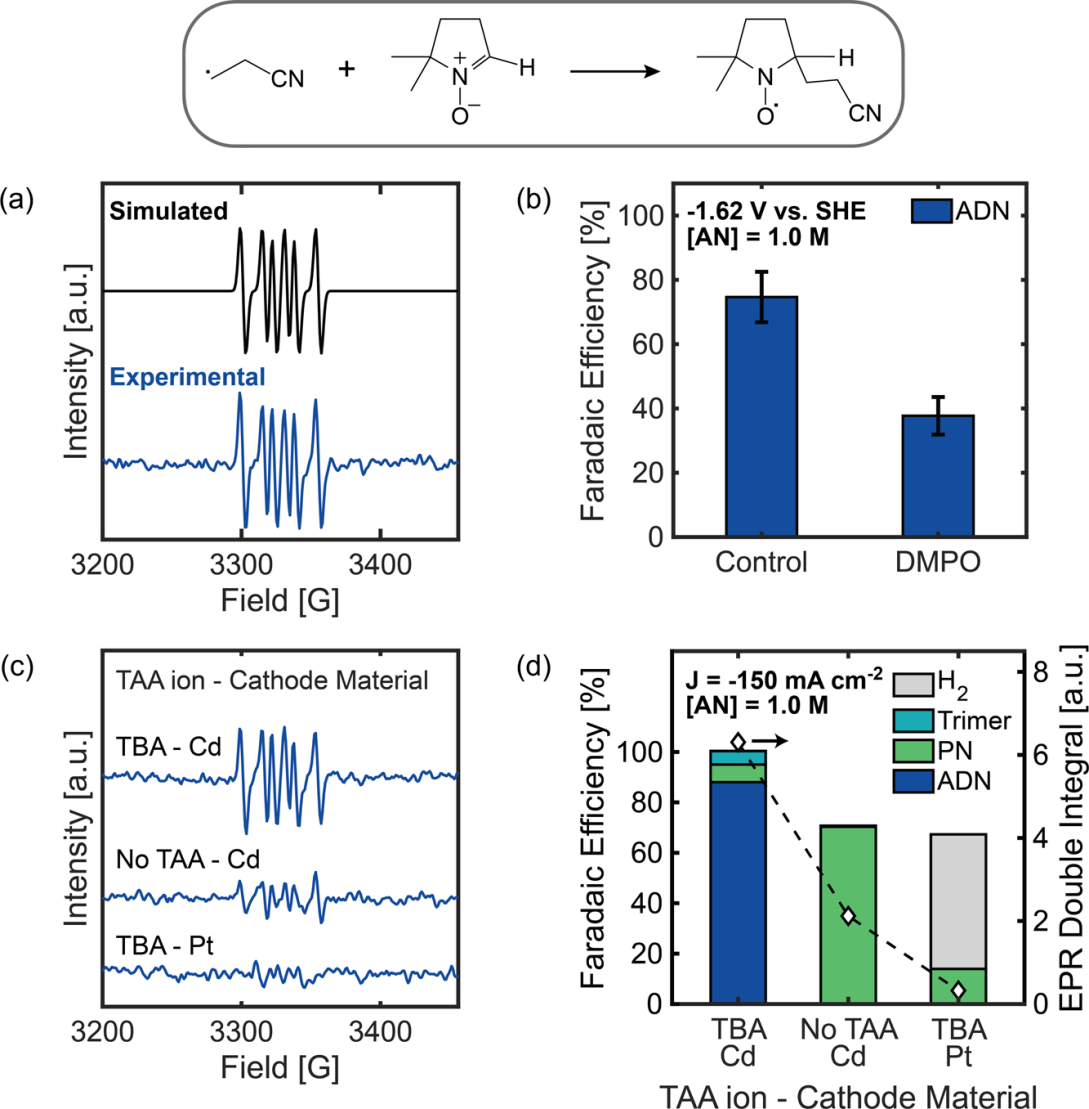
Capturing free radicals during acrylonitrile electroreduction. (a) Simulated and experimental EPR spectra instantly obtained after 20 min electrolysis on Cd foil at −150 mA cm^–2^. 50 mg DMPO as a spin trapper was added to the electrolyte after 5 min of electrolysis. (b) Comparison of the selectivity toward adiponitrile (ADN) for 10 min electrolysis on Cd foil at −1.62 V vs SHE between electrolytes with no spin trapper present (Control) and with excess of 0.45 M DMPO present acting as a spin trapper. Electrolytes contained 1 M AN, 0.5 M Na_3_PO_4_, 0.03 M EDTA, and 0.02 M TBA hydroxide. (c) EPR spectra instantly obtained after 10 min electrolysis at constant −150 mA cm^–2^ on the specified cathode material (Cd or Pt foil). The electrolyte contained 1 M AN, 0.28 M DMPO (320 mg), 0.5 M Na3PO4, 0.03 M EDTA, and 0 or 0.02 M TBA hydroxide (except for the no TAA experiment). (d) Electroreduction of acrylonitrile under three different conditions with their product distributions and relative concentration of free radicals given by the EPR double integral.

Since the first investigations on the electrodimerization of AN, there has been an ongoing debate regarding the mechanistic steps that lead to ADN production and whether radical intermediates exist in the solution. Previous studies have suggested that electrohydrodimerization of activated olefins occurs mainly through radical–radical coupling of two anion radicals followed by irreversible protonation,^76,77^ driven by a negligible coupling activation energy.^78^ However, there is limited experimental evidence of anion or alkyl radicals and whether they are present as surface-adsorbed species or as free radicals in solution. To provide insights into this question, we implemented spin-trapping techniques to examine the presence of free radicals in the dimerization mechanism. We implemented EPR spectroscopy techniques coupled with radical trapping to probe the presence of free radicals in the electrolyte. Since the produced alkyl radicals are short-lived, spin trap 5,5-dimethyl-1-pyrroline *N*-oxide (DMPO) was added to form a DMPO-radical stable adduct that is detectable using EPR. We ran electroreduction of AN on Cd foil under CP conditions at −150 mA cm^–2^ for 20 min, and DMPO was added to the electrolyte mixture after 5 min to allow for the accumulation of radicals. The presence of DMPO is not expected to kinetically affect the formation of the radical,^55,79^ which was confirmed by the almost identical LSV profiles with and without the presence of DMPO in the electrolyte (Figure S17). The EPR spectra collected upon completion of the reaction confirmed the presence of alkyl radicals (Figure 6a). The six observed peaks have close agreement with previously reported spin-adducts generated by the addition of DMPO to carbon-centered radical species,^80,81^ as well as with the simulated spectra of ^·^DMPO–CH_2_CH_2_CN. Control experiments showed no paramagnetic signal when no DMPO was added to the electrolyte mixture, and neither did an electrolyte-DMPO mixture that did not undergo electrolysis (Figure S18).

To further understand the role of free radicals, we performed a batch ADN electrosynthesis reaction with the presence of an excess spin trap (10-times DMPO as the maximum possible amount of electrochemically generated AN-radical) and contrasted it with a control AN electroreduction experiment. Both experiments were performed on a Cd rod at −1.62 V_SHE_ for 10 min. These potential conditions (−1.62 V vs SHE, −50 mA cm^–2^) were selected to maximize ADN formation, thereby making the effect of the radical trapping agent more evident. Figure 6b shows that the selectivity toward ADN significantly decreases from 75 to 38% when DMPO is added in excess, suggesting that the spin-traps reduce the presence of PN radicals and suppress the dimerization of AN. These results experimentally confirm that free radicals in the electrolyte are a key intermediate for ADN formation.

Having established the importance of free radicals, we next investigated how their production varies under different electrochemical conditions during AN electroreduction. We compared the relative magnitude of EPR signals under conditions favoring ADN formation (TBA ion in electrolyte, Cd cathode), PN production (no TAA ion in electrolyte, Cd cathode), and hydrogen evolution (TBA ion in electrolyte, Pt cathode). Figure 6c displays the EPR spectra for each condition, with experiments conducted using consistent total charge passed, DMPO concentration, and sample volume. A qualitative comparison of relative radical concentrations was achieved by double integrating the EPR signals (Figure S19). The product distributions and corresponding relative production of free radicals, shown in Figure 6d, confirm that ADN formation requires a relatively high concentration of free radicals for coupling. When PN formation is favored, free radicals are detected at lower concentrations, suggesting that AN hydrogenation primarily occurs as a surface reaction. Under conditions favoring hydrogen evolution, negligible radicals are detected due to the very low rates of AN reduction. These results demonstrate a correlation between the concentration of free radicals and the resulting product distribution in AN electroreduction.

Our experimental observation of free radicals during AN electroreduction suggests that given that the production of ADN heavily depends on the cathode material^31^ and that its reaction kinetics rely on the presence of TAA ions, ADN is likely formed through the generation of alkyl radicals that self-couple in the solution phase. This mechanistic insight and experimental investigation approach could be extended to related electro-organic synthesis processes, particularly electrohydrodimerization of other activated olefins.

## Conclusions

This study provided experimental insights into the role of the near-electrode microenvironment in controlling selectivity in AN electrohydrodimerization to ADN. In situ ATR-FTIR evidence suggests that TAA ions populate the EDL, with increasing local concentration at greater potentials, thus creating a microenvironment that favors interactions with organic molecules and enhances the concentration of AN while expelling water molecules from the electrode interface. Water molecules are displaced from near the electrode surface by TAA and alkali cations, and the extent of the displacement scales with the effective cation radii as supported by MD simulations. KIE and isotope incorporation studies validated that PN formation is rate-limited by a proton transfer, while the dimerization toward ADN is likely not. Thus, selective ADN formation can be accomplished when the proton transfer steps leading to PN and HER are hindered (e.g., adding TAA ions and operating at high pH). Lastly, radical trapping and EPR spectroscopy demonstrated the presence of free radicals during AN electroreduction, suggesting that coupling of PN radicals primarily occurs in the electrolyte. These experimental insights on near-electrode molecular processes support decades-old mechanistic hypotheses derived from empirical observations from electrolysis experiments. The results also provide thorough evidence of the complex interconnected molecular processes that control the selectivity of AN hydrodimerization and provide fundamental engineering guidance on the design of electrode/electrolyte microenvironments to develop high-performing electro-organic reactions. Fundamental questions regarding the role of surface adsorbed species (e.g., H or AN-derived species) on the reaction performance remain unanswered and will likely require investigations that deploy advanced in situ surface spectroscopy tools beyond those implemented in this study. Overall, the results highlight the importance of carefully controlling the electrical double layer (EDL) for selective organic electrosynthesis. These observations are crucial for designing optimal electrolyte solutions and electrode interfaces for organic electrosynthesis. The insights gained from this study not only enhance our understanding of ADN electrochemical synthesis but are also translatable to other emerging electro-organic synthesis processes–particularly those involving free radical intermediates – and have the potential to increase the number of electrochemical processes deployed in industry.

## Supporting Information Available

Technical details for each method, including used chemicals and materials, in situ ATR-FTIR electrochemical characterization, metal thin films deposition, electrochemical characterization in H-type cell, chemical quantification via GCMS and H^1^NMR, EPR measurements, and MD simulations (PDF)

## Abbreviations

ADN: adiponitrile
AN: acrylonitrile
ATR-FTIR: attenuated total reflectance Fourier transform infrared spectroscopy
CP: chronopotentiometry
DFT: density functional theory
DMPO: 5,5-dimethyl-1-pyrroline *N*-oxide
EDL: electrical double layer
EIS: electrochemical impedance spectroscopy
EPR: electron paramagnetic resonance
GC-MS: gas chromatography–mass spectrometry
HER: hydrogen evolution reaction
KIE: kinetic isotopic effects
LSV: linear sweep voltammetry
MD: molecular dynamics
OHP: outer Helmholtz plane
PN: propionitrile
TAA: tetraalkylammonium
TBA: tetrabutylammonium
TMA: tetramethylammonium
