# Supplementary material for: Molecular Processes That Control Organic Electrosynthesis in Near-Electrode Microenvironments
Source: J Am Chem Soc. 2025 Jan 27;147(5):4296–307. doi: 10.1021/jacs.4c14420 (PMC11803752; doi:10.1021/jacs.4c14420)
Supplement: Supplementary file 1 — ja4c14420_si_001.pdf [file ja4c14420_si_001.pdf]

Supporting Information

Molecular Processes that Control Organic Electrosynthesis in Near-Electrode Microenvironments

Ricardo Mathison,[a] Rasha Atwi,[b] Hannah B. McConnell,[a] Emilio Ochoa,[a] Elina Rani,[a] Toshihiro Akashige,[1] Jason A. Röhr,[a,c] André D. Taylor,[a] Claudia E. Avalos,[d] Eray S. Aydil,[a] Nav Nidhi Rajput,[b] Miguel A. Modestino[a]
[a] Department of Chemical and Biomolecular Engineering, New York University, Brooklyn, NY 11201, USA
[b] Department of Materials Science and Chemical Engineering, Stony Brook University, Stony Brook, NY 11794, USA
[c] General Engineering, New York University, Brooklyn, NY 11201, USA
[4] Department of Chemistry, New York University, New York, NY 10003, USA

Corresponding author: Miguel A. Modestino
Email: modestino@nyu.edu

This PDF file includes:
Figures S1 to S21
Tables S1 to S2

Experimental Methods

Chemicals and materials

Tetraethylammonium hydroxide (TEA, 40% wt. % in H_2O), tetrapropylammonium hydroxide (TPA, 40% wt. % in H_2O), acrylonitrile (AN, >99%), propionitrile (PN, 99%), adiponitrile (ADN, 99%), sodium phosphate (Na_3PO_4, 96%), potassium phosphate (K_3PO_4, ≥98%), rubidium hydroxide hydrate ($RbOH \cdot H_2O$), cesium hydroxide hydrate ($CsOH \cdot H_2O$, ≥90%, ≥99.5% metals basis), phosphoric acid (H_3PO_4, 85% wt. % in H_2O), deuterium oxide (D_2O, 99.8% atom % D), ethylenediaminetetraacetic acid disodium salt dihydrate ($EDTA \cdot 2H_2O$, ≥98.5%), sulfuric acid (H_2SO_4, 95-97%), heptane (≥99%), and platinum foil (0.125-0.135 mm thick, 99.9%) were purchased from Sigma Aldrich. Toluene was purchased from VWR. Tetramethylammonium hydroxide (TMA, 25% wt. % in H_2O), sodium formate (98%), and Cadmium foil (0.5 mm thick, 99.9975%) were purchased from Fisher Scientific. Tetrabutylammonium hydroxide (TBA, 40% wt. % in H_2O) was purchased from TCI Chemicals. 5,5-Dimethyl-1-Pyrroline-N-Oxide (DMPO, 97%) and hexane-1,3,6-tricarbonitrile (trimer, 98%) were purchased from Ambeed (IL, USA). Platinum gauze (52 mesh woven from 0.1 mm dia wire, 99.9%) was purchased from Alfa Aesar. Platinum rod (1.6 mm diameter and 31.2 mm length) was purchased from Analytical Sales and Services (NJ, USA). Nafion 117 was purchased from the Fuel Cell Store. Hydrogen calibration gases (310 ppm and 1000 ppm, balance air) were purchased from GASCO (IL, USA) and DOD Technologies (IL, USA), respectively. Argon gas was purchased from Airgas (PA, USA). Tetraakylammonium (TAA), rubidium, and cesium phosphate solutions were prepared by reacting their respective hydroxides with phosphoric acid stoichiometrically. The final pH of each solution was measured with a B30PCI pH meter from VWR. Deionized (DI) water was used for all experiments in this work.

In-situ ATR-FTIR electrochemical characterization

An electrochemical flow reactor was integrated with ATR-FTIR spectroscopy to probe near-electrode microenvironments during AN electroreduction. The internal reflection element (IRE) was a micro-grooved Si wafer (universal, 11 mm x 9 mm x 0.5 mm) placed on the specular reflection ATR accessory (20º angle of incidence) purchased from IRUBIS GmbH (München, Germany). Metal thin films were deposited using an Ångstrom thermal evaporator under a high vacuum (10^{-6} Torr) onto the wafer, which served as the working electrode for the electrochemical cell. A 7 nm Cr adhesion layer was first deposited at 0.2 Å s^{-1}, followed by a 5 nm Ag layer at 0.2 Å s^{-1}. Cr (99.998% purity) and Ag (99.99& purity) were purchased from Kurt J. Lesker. The undivided electrochemical flow cell was designed and fabricated using a Stratasys® Objet30 3D printer with VeroClear resin as printing material. The electrochemically active surface area was a circle of 0.2 cm^2 delimited by a Viton o-ring used to seal the cell. A platinum rod served as the counter electrode in all experiments with a surface area of 0.5 cm^2. The electrical connection with the thin working electrode was made with pogo pins, which were not in contact with the liquid electrolyte. Chronopotentiometry (CP) experiments were performed using a BioLogic SP-50 potentiostat.

A fresh aqueous solution of the desired TAA alkyl chain length, alkali cation size, and concentration of spectator ions and AN was prepared before each spectroelectrochemical experiment. A continuous flow of 0.32 mL/s of the electrolyte was recirculated from a reservoir using a Reglo ICC peristaltic pump (Ismatec®). A Nicolet iS50 FTIR Spectrometer and OMNIC software were used for spectral data collection. Multiple spectra were obtained for each current density and/or electrolyte composition to allow the near-electrode microenvironment to reach a pseudo-steady state. A minimum of 25 spectral scans were averaged using 4 cm^{-1} resolution. All data presented in this study were obtained using the same air or DI water background. The reactor was emptied and rinsed with water for at least a minute every time there was a change in electrolyte composition. The IR absorption peaks of relevant molecules are summarized in Table S1. CP experiments were performed at a current density of -25 mA

cm^{-2} based on the observation of TAA ion saturation at the interface at this value. Higher current densities were not explored due to potential delamination of the thin metal electrodes and the lack of expected changes in local TAA ion concentration above this threshold.

Electrochemical characterization in H-type cell

A 3-electrode setup was used to study the kinetic isotope effect (KIE) on the cathodic half-cell reactions. Electrochemical Impedance Spectroscopy (EIS) and CP experiments were performed using a BioLogic VSP-300 potentiostat equipped with a ±1 A/±48 V booster. CP techniques were performed for 10 minutes, yielding AN conversions under 25%. A machined Teflon H-cell was separated with a Nafion N117 membrane and sealed between 2 Viton gaskets, avoiding the deposition of metal ions from the anode on the cathode surface. The electrodes consisted of cadmium foil of 1 cm^2 as the cathode, a platinum mesh as the anode, and an Ag/AgCl electrode in saturated KCl solution as the reference electrode. The cadmium electrode was stored in an aqueous electrolyte whenever not in use. Electrodes not stored in this way could affect the reactor performance, possibly due to the formation of undesired surface oxides. EIS was used to characterize the resistance of the cell and correct the electrode potential for iR losses. Before each experiment, two stock solutions of 0.5 M Na3PO4, 0.03 M EDTA, and 0.02 M TBA-OH in H_2O and D_2O were prepared and mixed with AN in the desired electrolyte D_2O fraction and AN concentration. A constant catholyte volume of 7 mL was vigorously stirred with 4x8 mm micro-stir bars. The anolyte consisted of a 1 M solution of sulfuric acid.
Free radical capture experiments were performed on the same three-electrode system. CP techniques were performed for 10 minutes on the machined Teflon H-cell used for KIE studies. The electrodes consisted of a cadmium rod of 1 cm^2 as the cathode, a platinum mesh as the anode, and an Ag/AgCl electrode in saturated KCl solution as the reference electrode. Before each experiment, a fresh stock solution of 0.5 M Na_3PO_4, 0.03 M EDTA, and 0.02 M TBA-OH in H_2O was mixed with AN. The anolyte consisted of a 1 M solution of sulfuric acid.
Control electrolysis experiments for free radical production comparison were performed in a machined Teflon H-cell with the cathode side sealed to the atmosphere and equipped with an argon purge to aid in the collection of gas products. A Nafion 117 membrane separated the cathode and anode sides. Argon flowed at a rate of 26 sccm into the reactor. A cold trap was incorporated downstream of the argon to condense volatile products. Gas products were collected using a Tedlar sample bag. Platinum mesh served as the anode, and an Ag/AgCl electrode in saturated KCl solution was used as the reference electrode. A constant electrolyte volume of 10 mL was used, and the cathode surface area was 3 cm^2.

Chemical quantification

Product mixtures from KIE and control electrolysis experiments for free radical production comparison were analyzed using the following procedure. After each experiment, the aqueous electrolyte mixture was added to a separatory funnel and mixed with toluene for liquid-liquid extraction of the organic molecules (50 μL of heptane was added as an internal standard). The organic phase was then analyzed using a Shimadzu gas chromatographer equipped with a mass spectrometer (GCMS-QP2010). Figure S20 shows the calibration curves for ADN and PN in electrolytes used to quantify the reaction products. Products obtained from free radical capture experiments under the presence of excess spin trap were analyzed via H^1NMR using a Bruker Avance III 400 NMR Spectrometer. Spectra was obtained with a delay time of 8 s, 16 scans per measurement, and without solvent suppression. The samples consisted of 70% catholyte and 30% D_2O, with the addition of a solution of sodium formate for product quantification. See Figure S21 for sample spectra.

Hydrogen gas quantification in control experiments was performed using an Agilent 990 Micro GC equipped with a MolSieve 5A column. Acquisition parameters were: injection temperature 110 °C,

injection time 40 ms, column temperature 80 °C, argon carrier gas, run time 80 s. A standard gas mixture containing H_2 was used as a reference.

Electron Paramagnetic Resonance (EPR) measurements

The following electrochemical procedure was used to capture and detect the free radicals generated during the electroreduction of AN carried out in an aqueous electrolyte. Electrolysis was carried out at -150 mA cm^{-2} to achieve detectable concentrations of radical species for EPR measurements and to access conditions where electrolyte composition and electrode material significantly impacted product distribution. During the electrolysis, 50 mg of DMPO was added to the electrolyte 5 minutes after the start of the reaction. After the electrolysis, an electrolyte sample of constant volume was extracted with a quartz capillary for immediate EPR tests. A qualitative assessment of free radical production under different electrochemical conditions was performed by collecting a sample after 10-minute electrolysis with 320 mg of DMPO. The liquids were analyzed on a quartz capillary tube at a constant sample volume of 25 μL. The EPR experiments were performed through a Bruker ELEXSYS E500 EPR. Eight accumulated scans were acquired to measure DMPO adducts. The experimental EPR spectra were simulated and fitted on Bruker Xenon.

Molecular dynamics (MD) simulations

MD simulations were performed on the 0.6 M AN, 0.5 M K_3PO_4, and 0.05 M TAA-OH (TBA, TPA, TEA, and TPA) aqueous electrolytes. The density of these electrolytes is 1.085 g cm^{-3}. MISPR[1] version 0.0.4 was utilized for automating the following steps. First, initial configurations were prepared by packing molecules in a 9×9×9 nm^3 box using PACKMOL.[2] GAFF[3] parameters were used for all electrolyte species, except for water which was described by the rigid SPC/E[4] water potential. The constraints for the rigid water model were fulfilled using the SHAKE[5] algorithm. K^+ nonbonded parameters were obtained from ref[6]. Ionic charges were scaled by a factor of 0.75 to account for electronic polarization effects implicitly[7]. Lennard–Jones interactions were truncated at a cutoff distance of 1.2 nm. The particle–particle particle-mesh (PPPM)[8] method was used to handle long-range electrostatic interactions using a cutoff of 1.2 nm. NPT simulations were performed for 2 ns with the Nosé/Hoover[9, 10] thermostat and barostat. The systems were melted to 227 ℃ for 2 ns and quenched in three steps to achieve a temperature of 25 ℃ and ensure that the molecules are not trapped in the metastable state. NVT simulations were performed for 10 ns and molecular trajectories were sampled every 50 ps, resulting in 200 configurations per system, from which the radial distribution functions, solvation structures, and diffusion coefficients were calculated. The Einstein relation was used for diffusion coefficients, with the mean square displacements obtained using time intervals in which the electrolytes exhibit diffusive regime. To assess differences in the mobility of species of the same type, displacements were calculated in 2 ns intervals averaged over all possible choices of the time interval of the NVT trajectory. Distributions of diffusion coefficients of individual species were then computed from the displacements and fit using Gaussian kernel density estimation (Figure S14).

Bulk Electrolysis Product Distribution

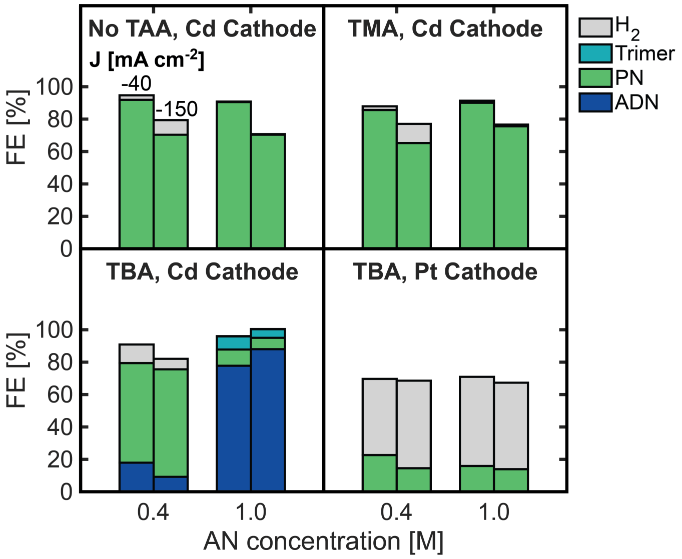

Figure S1. Product distributions for H-cell electrolysis. Selectivities towards adiponitrile (ADN), propionitrile (PN), hexane-1,3,6-tricarbonitrile (Trimer), and hydrogen gas (H_2), given by Faradaic efficiency (FE) for constant current electroreduction of AN in a divided reactor. The total charge transferred was kept constant at 3000 mA min. Each set of experiments consisted of a combination of TAA ion in the electrolyte (No TAA, TMA, or TBA) and cathode material (Cd foil or Pt foil) for two current densities and AN concentrations. Electrolytes contained 1 M AN, 0.5 M Na_3PO_4, 0.03 M EDTA, and 0.02 M TAA hydroxide (except for No TAA).

FTIR Baseline Correction algorithm

A baseline correction algorithm was developed to compare FTIR spectra with different baseline shifts. Varying the cathodic current in all experiments led to an upward shift in the spectra baseline at wavenumbers below 3000 cm^{-1}. The same deviation was observed when applying cell potentials greater than 1.5 V with a sample of distilled water. The baseline shift was intensified with increasing potentials until reaching a plateau at cell potentials of approximately 2.0 V. These shifts are attributed to polarization effects on thin metal films, influencing the interaction between incident infrared radiation and collective electron oscillations at the electrode surface.[11] The phenomenon depends on factors such as metal film thickness, applied potential, and polarization state of incident radiation, significantly affecting the optical properties of the metal-electrolyte interface.[12] Since this study focuses on the behavior of organic on aqueous electrolytes at potentials of interest (cell potentials > 1.5), baseline shifts are inevitable. A step-by-step workflow of the baseline-correction algorithm is as follows, and is provided in a GitHub repository.[13] Figure S2 shows the raw spectrum, polynomial fits, the predicted baseline, and the corrected spectrum for an example measurement.

1. **Baseline shift observed at cathodic currents**. The polarized cathode surface shifted the baseline, making it challenging to quantitatively assess changes in peak intensities as a function of the current density.
2. **Polynomial fit**. Two third-degree polynomials were fit to the baseline, one on each side of the peak(s) of interest.
3. **Predicted baseline**. A sigmoidal average was calculated between the two polynomials, using an S-shaped weighting function to smoothly transition from one polynomial to the other across the data range
4. **Correction**. The predicted baseline was subtracted from the raw spectra, giving a clean spectrum with deconvoluted peaks.
5. **Compare and analyze**. The corrected spectra were examined for the corrected baseline's flatness for accurate comparison at different applied current densities.

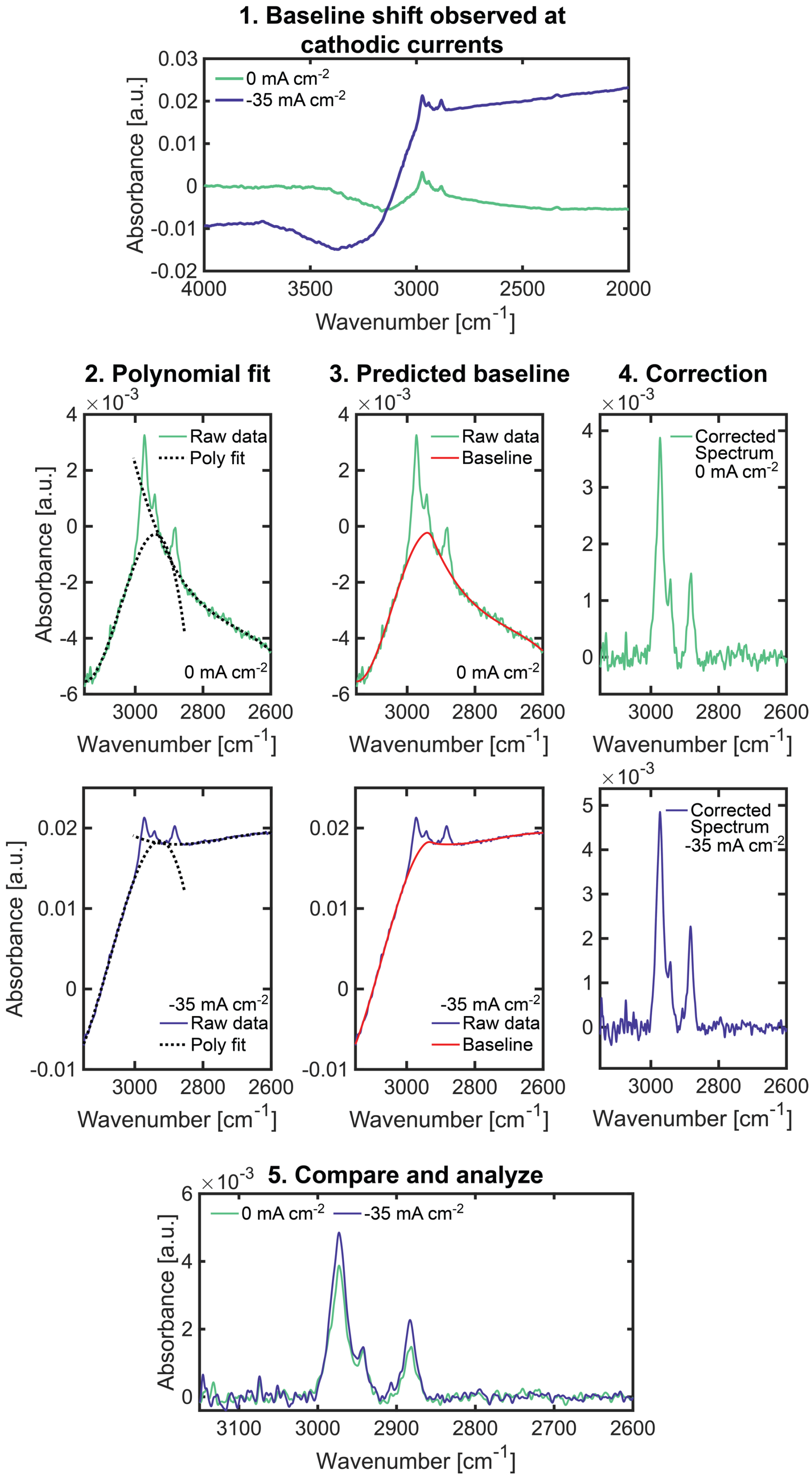

Figure S2. Baseline-correction algorithm for FTIR peak deconvolution at different applied current densities.

To ensure our baseline correction algorithm accurately preserves peak shapes and intensities while removing baseline shifts, we systematically evaluated its performance across a range of current densities. Figure S3a shows raw FTIR spectra collected at different cathodic current densities, where increasing current leads to progressively larger baseline shifts for wavenumbers below 3000 cm^{-1}. To validate our baseline correction approach, we compared our algorithm's predicted baseline against a more commonly used tangent reference line (Figure S3b). This tangent line was constructed by connecting the local minima on either side of the symmetric CH_3 stretch of TBA, the key peak used throughout this study. The area between this reference line and our predicted baseline (orange shading) was compared to the peak area above the predicted baseline (blue shading) across all tested current densities. Figure S3c demonstrates that while the total peak area (peak + residual) increases with current density and plateaus above 25 mA cm^{-2}, this trend is effectively captured by the corrected peak area alone. Importantly, the residual area between the reference line and predicted baseline remains relatively constant and substantially smaller than the peak area across all current densities, confirming that our algorithm successfully distinguishes peak intensity changes from baseline effects.

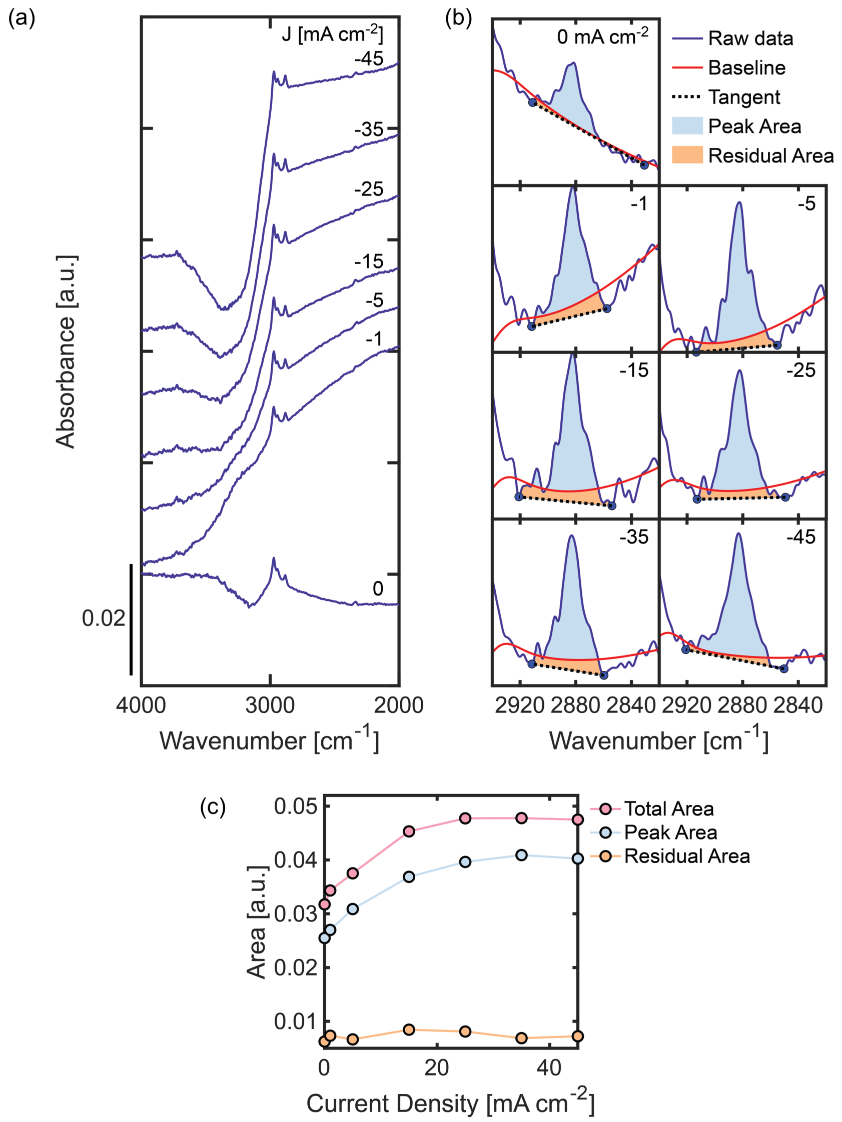

Figure S3. Validation of baseline-correction algorithm for FTIR peak deconvolution.

In situ ATR-FTIR Supporting Data

Table S1. IR absorption peaks of relevant molecules in this study.

Species	Wavenumber [cm^{-1}]	Vibration type	Assignment
H_2O	3000–3500	stretch	O–H
	1550-1750	bend	O–H
AN	2236	stretch	C≡N
TBA^+	2971	asymmetric stretch	C–H of CH_3
	2941	asymmetric stretch	C–H of CH_2
	2883	symmetric stretch	C–H of CH_3
	2847	symmetric stretch	C–H of CH_2
PO_4^{3-}	900-1100	stretch	P=O

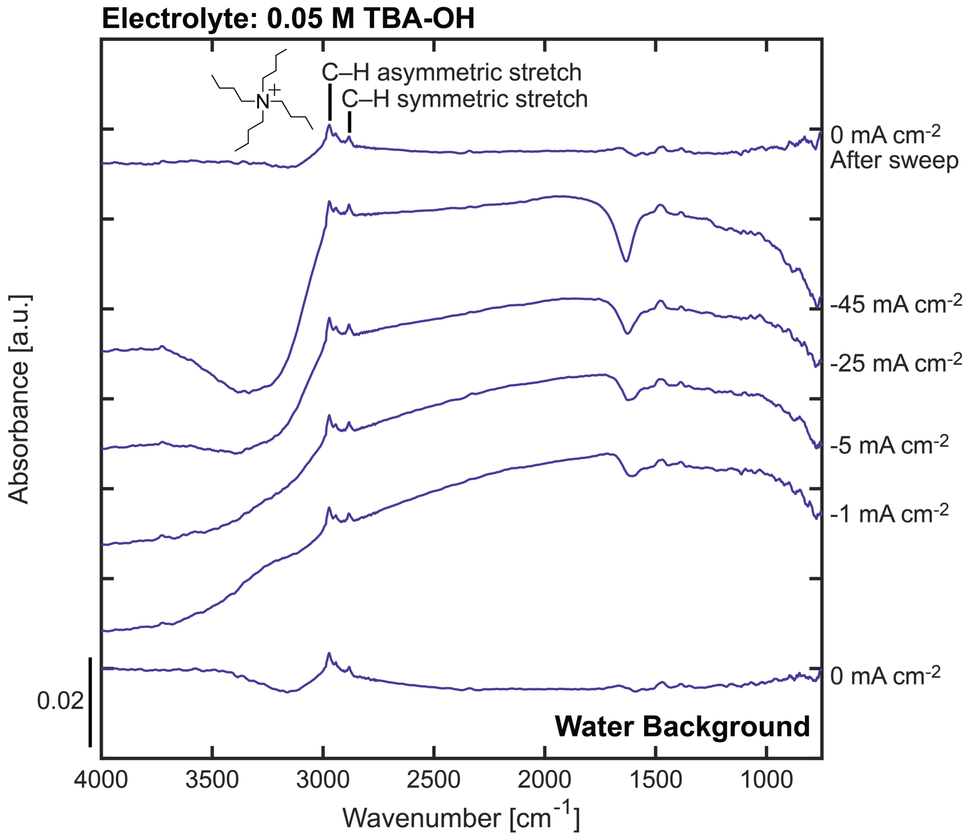

Figure S4. Full range FTIR spectra at different applied current densities. Spectra are represented as differences with respect to a DI water background spectrum. The electrolyte contained 0.05 M TBA hydroxide.

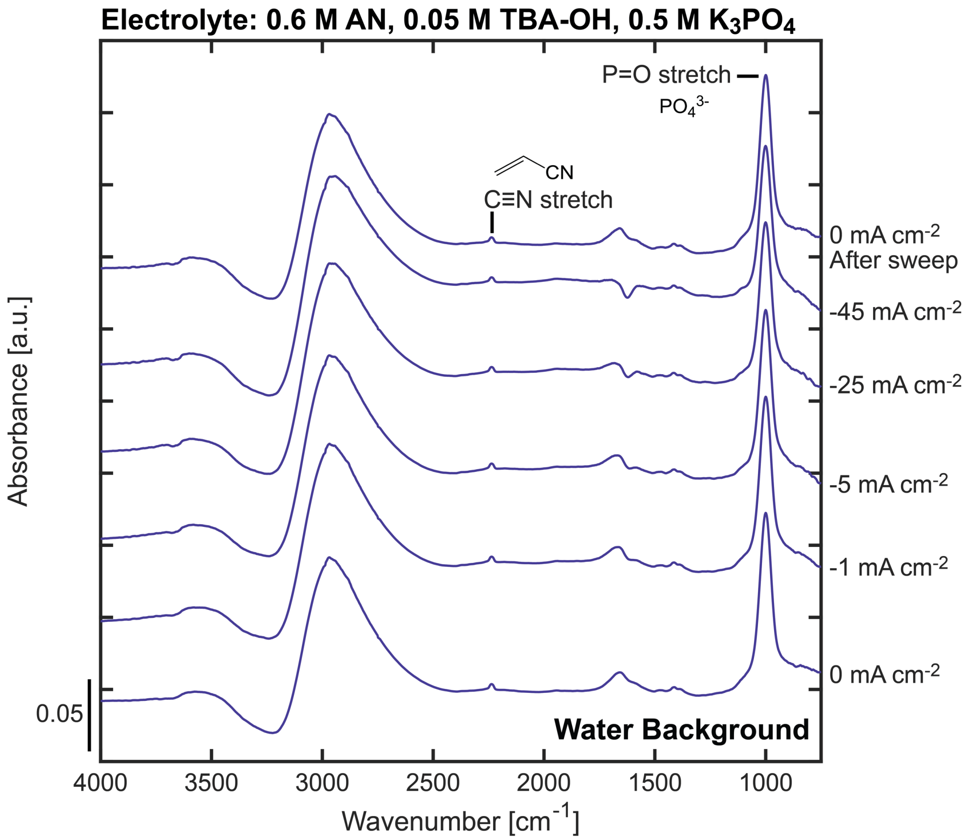

Figure S5. Full range FTIR spectra at different applied current densities. Spectra are represented as differences with respect to a DI water background spectrum. The electrolyte contained 0.6 M AN, 0.05 M TBA hydroxide, and 0.5 M K_3PO_4.

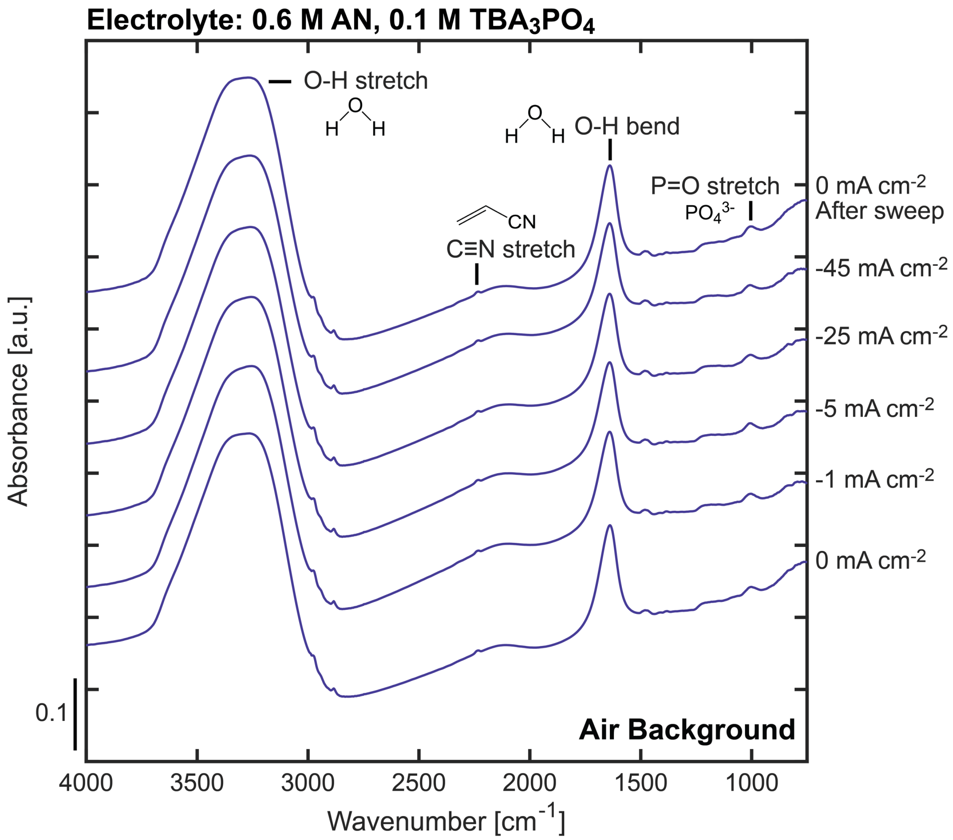

Figure S6. Full range FTIR spectra at different applied current densities. Spectra are represented as differences with respect to an air spectrum. The electrolyte contained 0.6 M AN and 0.1 M TBA phosphate.

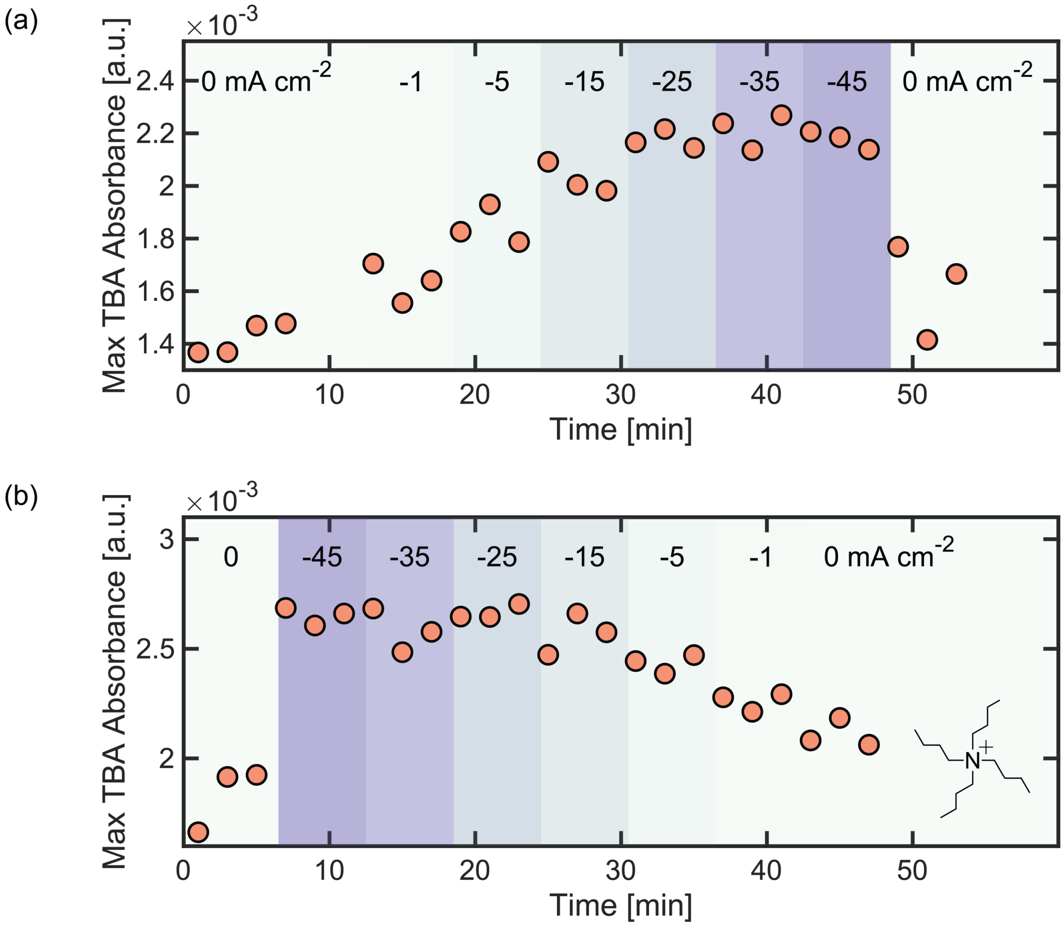

Figure S7. TBA near-electrode concentration under varying current densities in ascending and descending order. Time-progress of the maximum TBA absorbance in the chronological order in which the spectra were obtained in (a) ascending and (b) descending current density. TBA absorbance is depicted by the maximum absorbance of the symmetric CH_3 stretch. The electrolyte contained 0.05 M TBA hydroxide.

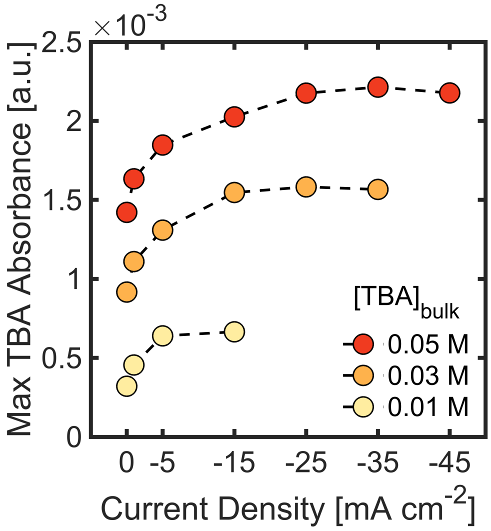

Figure S8. Effect of current density on TBA near-electrode concentration varying TBA bulk concentration. Effect of applied cathodic current on the near-electrode concentration of TBA ions depicted by the maximum absorbance of the symmetric CH_3 stretch. Multiple spectra were obtained for each experimental condition to confirm that the local concentration approached steady-state. The electrolyte contained TBA hydroxide.

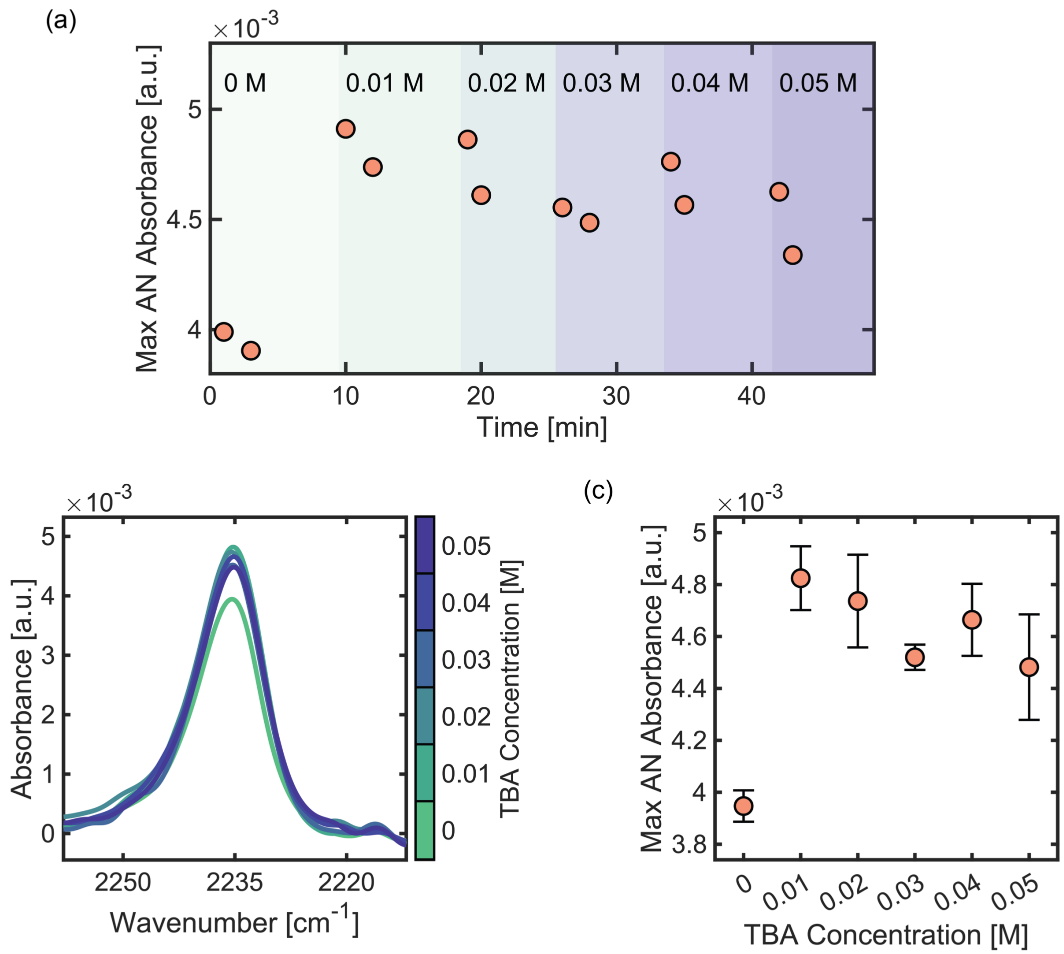

Figure S9. Effect of TBA ion concentration on AN near-electrode concentration. Effect of TBA ion bulk concentration on the near-electrode concentration of AN depicted by the maximum absorbance of the C≡N stretch. The electrolyte contained 0.6 M AN, 0.5 M K_3PO_4, and the TBA hydroxide concentration shown above. The current density was -25 mA cm^{-2}. (a) Time-progress of the maximum AN absorbance in the chronological order in which the spectra were obtained. (b) IR spectra of near-electrode AN as a function of TBA concentration. (c) Mean maximum AN absorbance for each experimental condition.

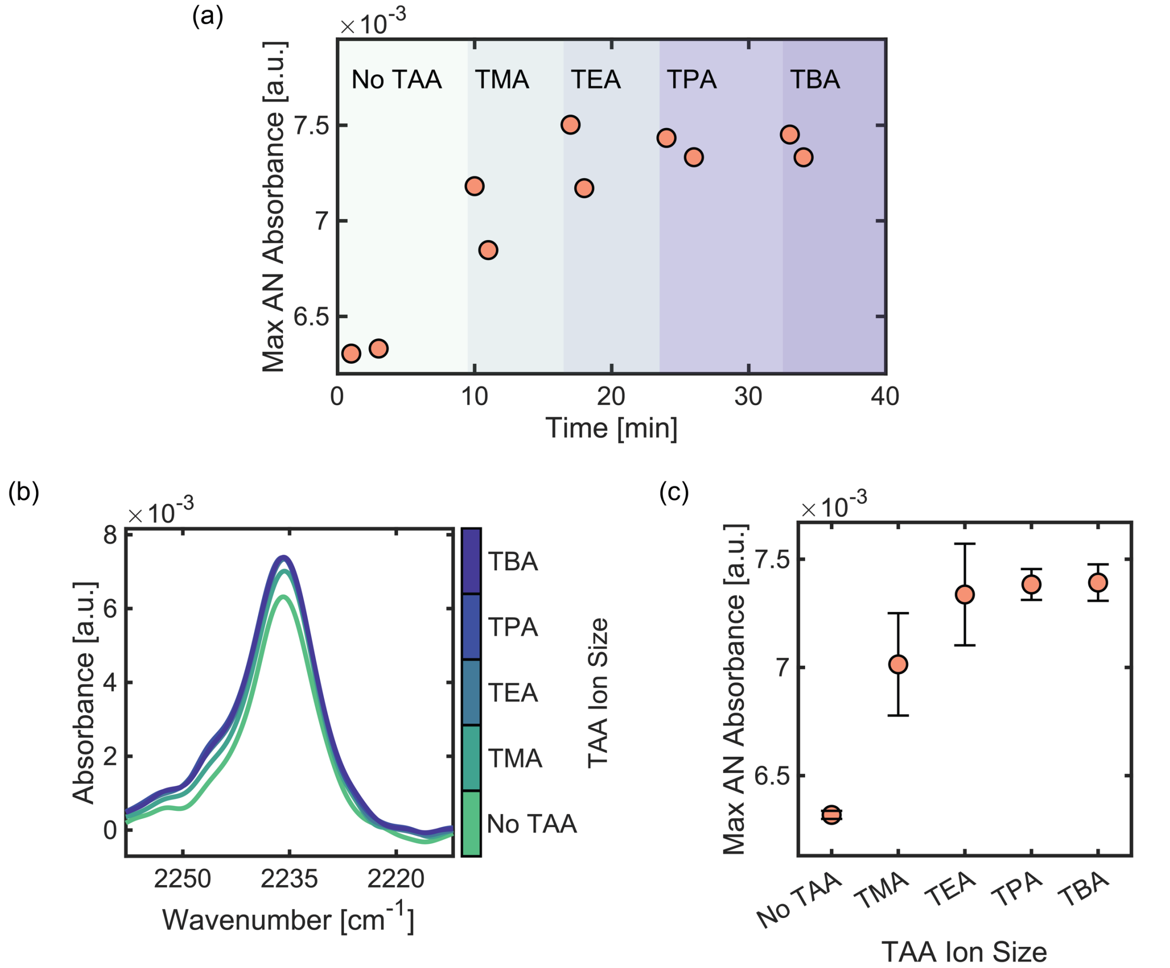

Figure S10. Effect of TAA ion size on AN near-electrode concentration. Effect of TAA ion size on the near-electrode concentration of AN depicted by the maximum absorbance of the C≡N stretch. The electrolyte contained 0.6 M AN, 0.5 M K_3PO_4, and 0.04 M TAA hydroxide (except for No TAA). The current density was -25 mA cm^{-2}. (a) Time-progress of the maximum AN absorbance in the chronological order in which the spectra were obtained. (b) IR spectra of near-electrode AN as a function of TAA ion size. (c) Mean maximum AN absorbance for each experimental condition.

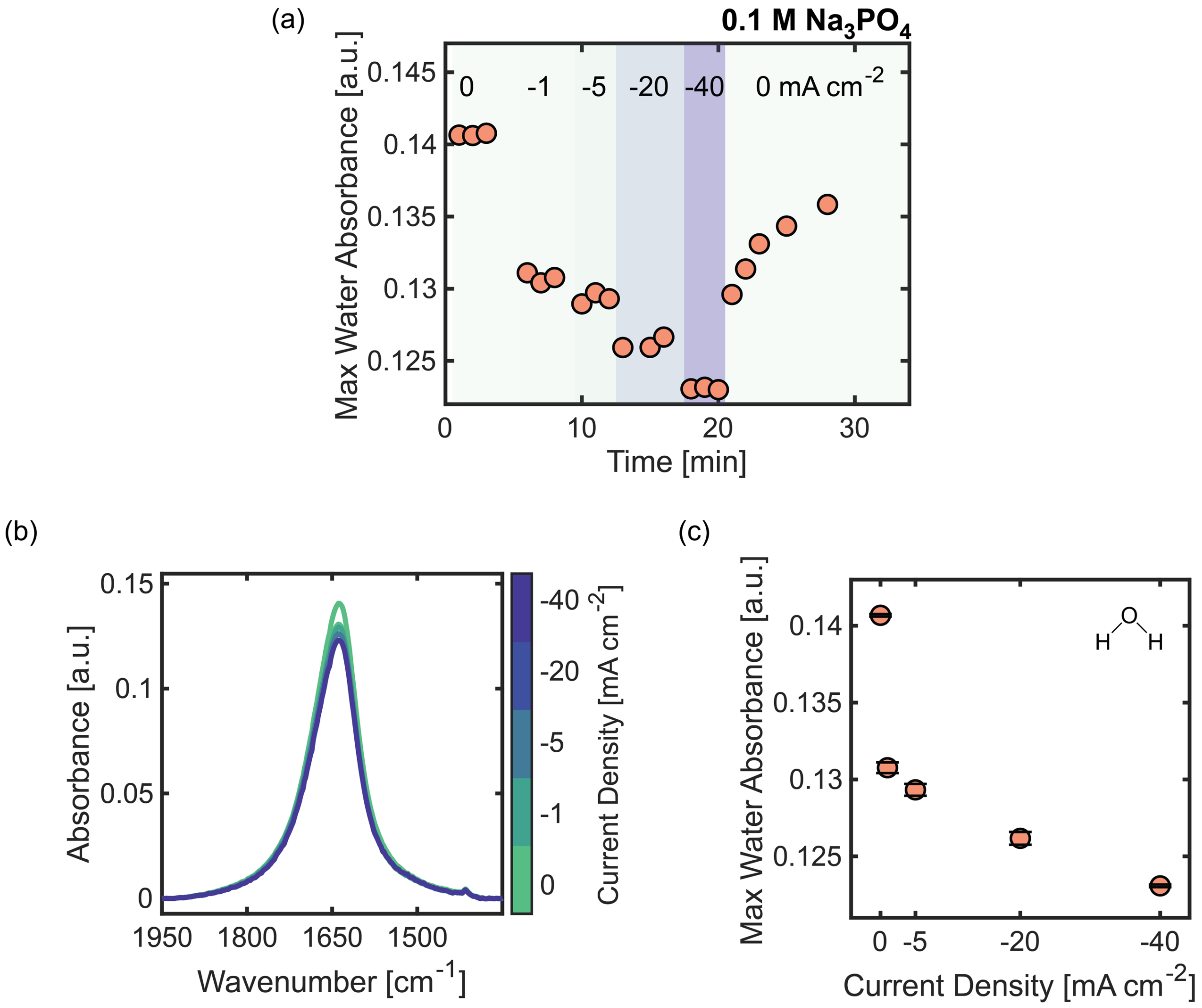

Figure S11. Effect of current density on water near-electrode concentration. Effect of current density on the near-electrode concentration of water depicted by the maximum absorbance of the O–H scissor. The electrolyte contained 0.6 M AN, 0.1 M Na_3PO_4, and the current density was varied from 0 to -45 mA cm^{-2}. (a) Time-progress of the maximum water absorbance in the chronological order in which the spectra were obtained. (b) IR spectra of near-electrode water as a function of current density. (c) Mean maximum water absorbance for each experimental condition.

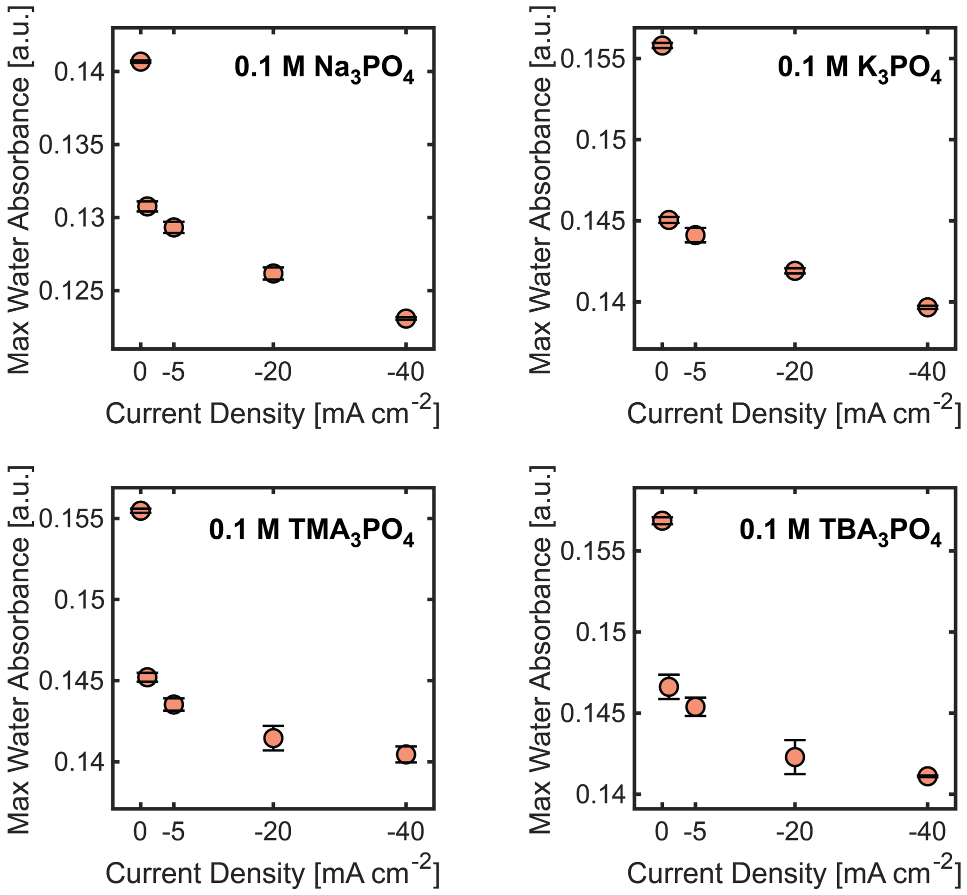

Figure S12. Effect of current density on water near-electrode concentration under varying electrolyte cation. Local water concentration as a function of applied cathodic currents from 0 to -45 mA cm^{-2} for four different electrolyte cations: Na, K, TMA, and TBA. For all experiments, the local concentration of water is depicted by the maximum absorbance of the O–H scissor, and multiple spectra were obtained for each experimental condition to confirm that the local concentration approached steady-state. The electrolyte contained 0.1 M $(Cation)_3PO_4$ and 0.6 M AN.

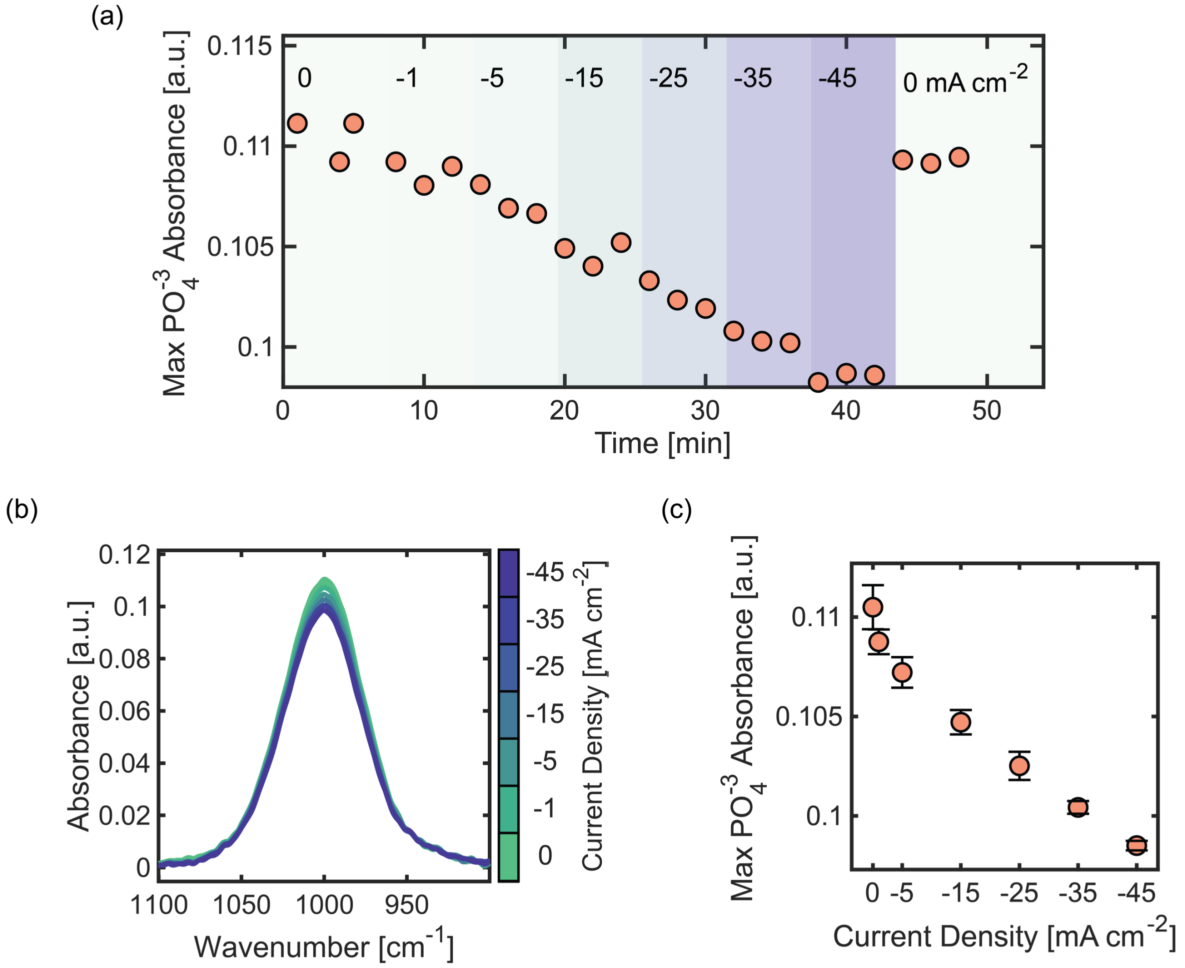

Figure S13. Effect of current density on phosphate ion near-electrode concentration. Effect of current density on the near-electrode concentration of phosphate depicted by the maximum absorbance of the P=O stretch. The electrolyte contained 0.6 M AN, 0.5 M K_3PO_4, and 0.05 M TBA hydroxide. The current density was varied from 0 to -45 mA cm^{-2}. (a) Time-progress of the maximum phosphate absorbance in the chronological order in which the spectra were obtained. (b) IR spectra of near-electrode phosphate as a function of current density. (c) Mean maximum phosphate absorbance for each experimental condition.

Diffusion coefficients computation of AN and TAA in water

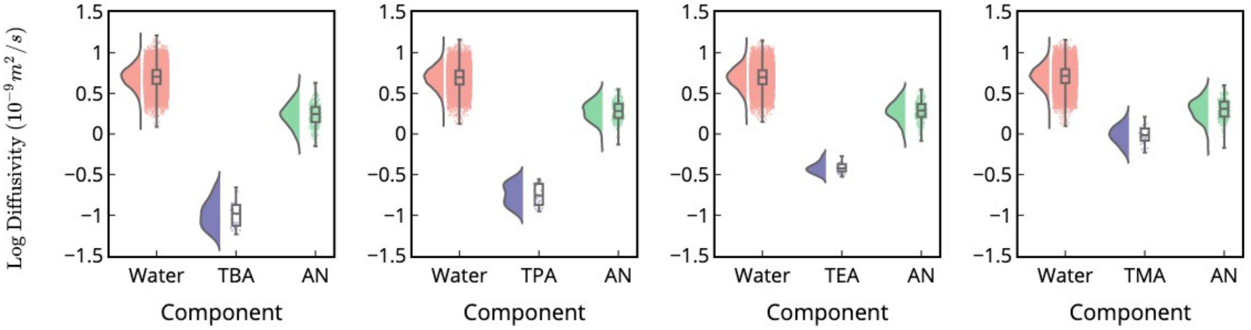

Figure S14. Raincloud plots (i.e. combined violin, box, and strip plots) for the MD-computed diffusion coefficients of water, TAA ions, and AN. The strip plots show all the diffusion data for each specie. In the box plots, the whisker tails correspond to the extrema, the boundaries correspond to the interquartile range, and the horizontal lines correspond to the median. The violin plots show the probability density of the diffusion coefficients of each electrolyte component.

Electrochemical characterization of AN reduction with H/D solvents

Reported working electrode potentials accounted for the iR compensation, and reported values were calculated according to the following equation:

$$E_{WE} = E_{vs.Ag/AgCl} + E_{RE} - iR_{comp} \quad (1)$$

where E_{WE} and E_{RE} correspond to the potential at the working and reference electrode, respectively, $E_{vs.Ag/AgCl}$ is the measured potential, i is the set current, and R is the electrolyte resistance.

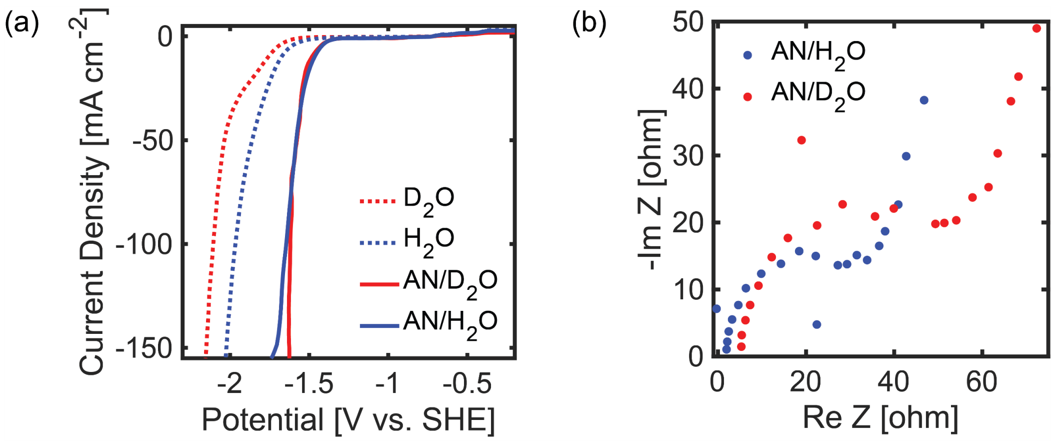

Figure S15. Linear sweep voltammetry of acrylonitrile electroreduction with H/D solvents. (a) IR-compensated linear sweep voltammetry curves on Cd foil with (solid line) and without (dotted lines) 1 M acrylonitrile dissolved in D_2O (red lines) and H_2O (blue lines). (b) Nyquist plots for (7 mV sinus amplitude) for 1 M AN solutions in D_2O and H_2O electrolytes. All experiments contained 0.5 M Na_3PO_4, 0.03 M EDTA, and 0.02 M TBA hydroxide.

Isotopic incorporation studies

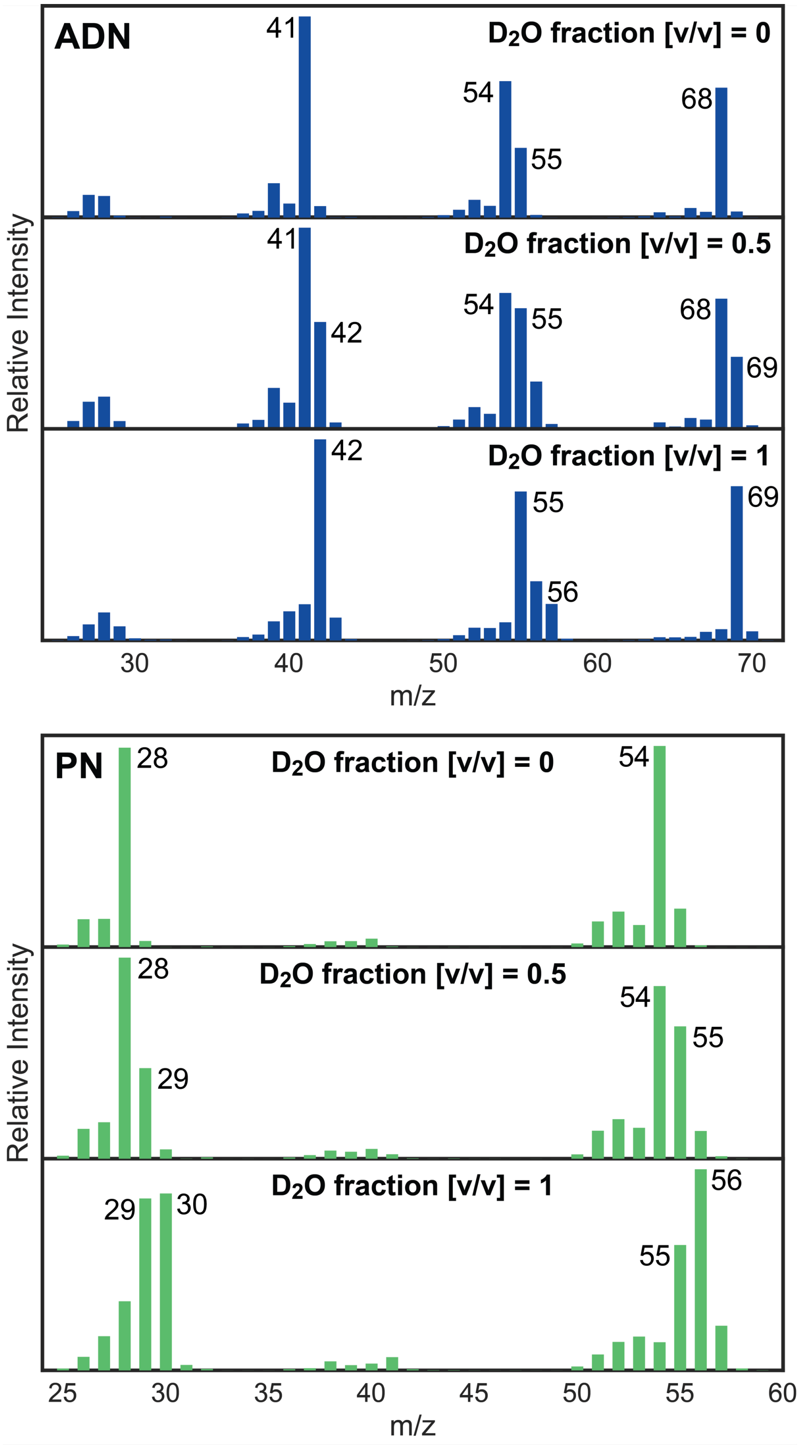

Figure S16. Mass spectra of acrylonitrile electroreduction products. Mass spectra produced from 10-minute electrolysis on Cd foil at -1.77 V vs. SHE with varying H/D isotopic compositions in the solvent depicted by D_2O volume fraction in the solvent with H_2O as the remaining solvent for adiponitrile (ADN) and propionitrile (PN).

Fragmentation patterns of ADN (0-D)

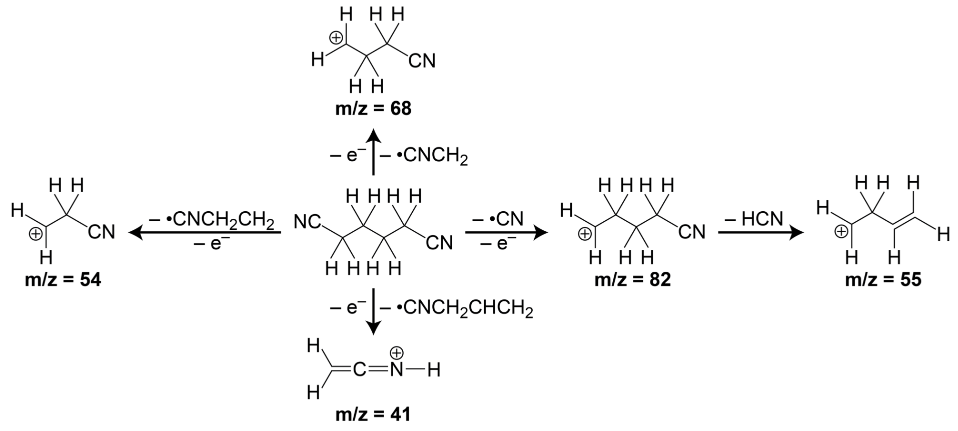

Fragmentation patterns of ADN (1-D)

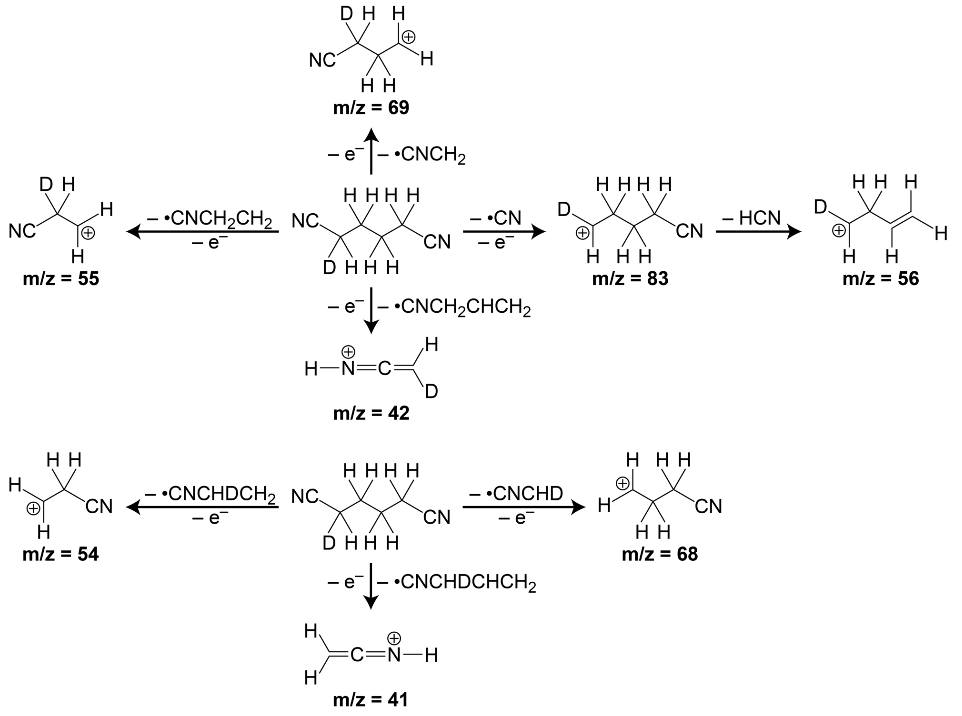

Fragmentation patterns of ADN (2-D)

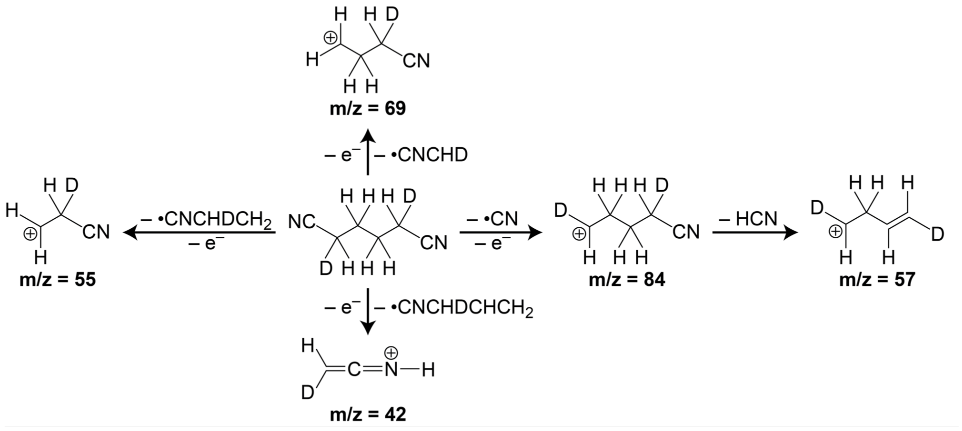

Fragmentation patterns of PN (0-D)

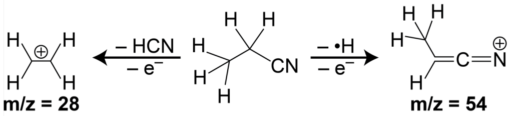

Fragmentation patterns of PN (1-D)

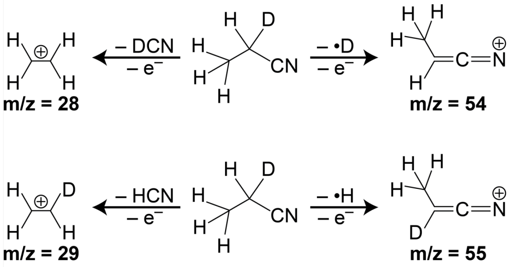

Fragmentation patterns of PN (2-D)

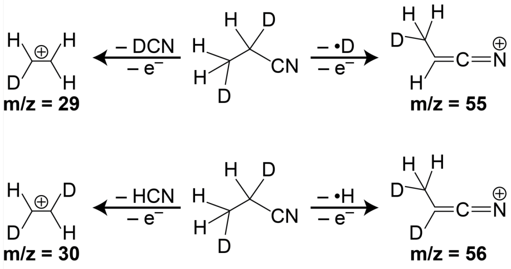

The fraction of H/D in ADN and PN were calculated by fitting two parameters (f_H and f_D) to mass spectroscopy experimental data as shown in the following equation:

$$MS_i = f_H \cdot MS_H + f_D \cdot MS_D \quad (2)$$

where MS_i, MS_H, and MS_D are the mass spectra of the molecule of interest – a matrix of m/z values and their corresponding relative abundance – for the solvent mixture being calculated (i), obtained in an H-only electrolyte (H), and obtained in a D-only electrolyte (D). Parameters f_H and f_D are fitted in the equation using the fmincon function in MATLAB. The accuracy of this model is tested by adding f_H and f_D, which theoretically must be 1. Results from the model are shown in Table S2.

Table S2. Isotopic incorporation studies results.

Molecule	D_2O Fraction	f_H	f_D	$f_H + f_D$
ADN	0.25	0.833	0.162	0.995
	0.50	0.637	0.366	1.003
	0.75	0.366	0.628	0.994
PN	0.25	0.841	0.157	0.998
	0.50	0.651	0.335	0.986
	0.75	0.404	0.582	0.986

Control experiments of radical capture and detection

Additional electron paramagnetic resonance (EPR) spectroscopy measurements were performed to confirm the capture of electrochemically generated carbon-centered radicals (Figure S18). No radical was detected in the electrolyte mixture before reaction with or without the presence of spin trap DMPO (a-b). No radical was detected when there was no spin trap DMPO present during electrolysis, given by the short-lived nature of carbon-centered radicals produced (c). Electrochemical reaction parameters were varied to assess the relative magnitude of EPR signal for conditions that favor adiponitrile (d), propionitrile (e), and hydrogen (f). Measurements shown in Figure S18d-f were performed with a consistent total charge passed (270 C), reaction volume (10 mL), concentration of AN and DMPO (1 and 0.28 M), and volume collected for analysis (25 μL). The relative concentration of free radicals in Figure S18d-f was assessed by performing double integration of the EPR signal,[14] as shown in Figure S19.

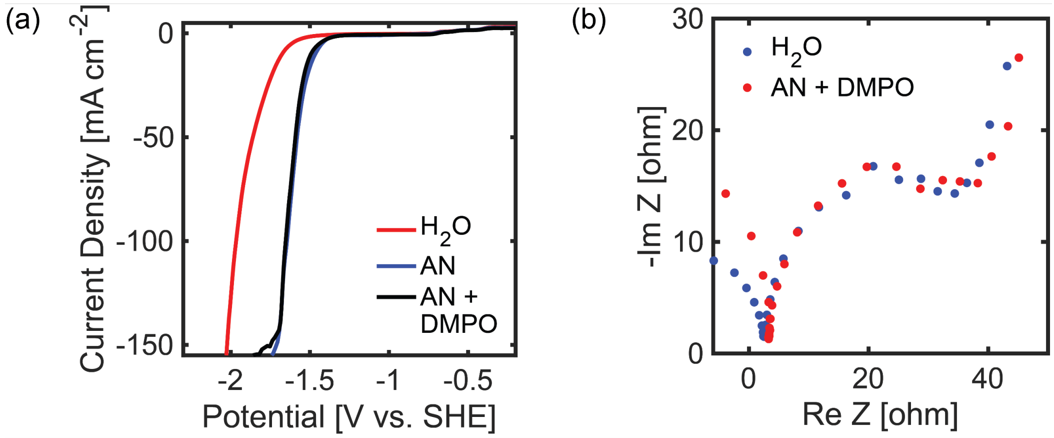

Figure S17. Linear sweep voltammetry of acrylonitrile electroreduction with a spin trapper. (a) IR-compensated linear sweep voltammetry curves on Cd rod in electrolyte (red line), 1 M AN in electrolyte (blue line), and 1 M AN and 0.45 M DMPO in electrolyte (black line). (b) Nyquist plots for (7 mV sinus amplitude) for electrolyte and 1 M AN and 0.45 M DMPO in electrolyte. All experiments contained 0.5 M Na_3PO_4, 0.03 M EDTA, and 0.02 M TBA hydroxide.

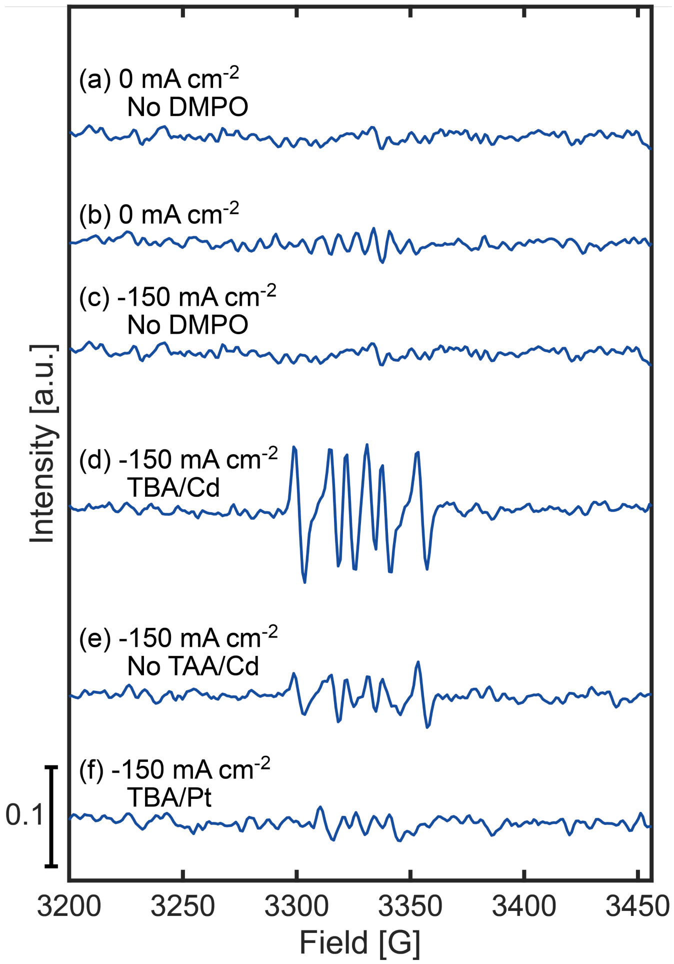

Figure S18. Electron paramagnetic resonance measurements. Experiments were performed with fresh solutions collected right after 10-minute electrolysis for those with non-zero current densities. The liquids were analyzed on a quartz capillary tube at a constant sample volume of 25 μL. The electrolyte contained 1 M AN, 0.28 M DMPO (320 mg), 0.5 M Na_3PO_4, 0.03 M EDTA, and 0.02 M TBA hydroxide unless specified otherwise. (a) No reaction, no DMPO present in the electrolyte. (b) No reaction. (c) Reaction at -150 mA cm^{-2} on Cd foil as the cathode, no DMPO present in the electrolyte. (d) Reaction at -150 mA cm^{-2} on Cd foil as the cathode. (e) Reaction at -150 mA cm^{-2} on Cd foil as the cathode, no tetraakylammonium ions present in the electrolyte. (f) Reaction at -150 mA cm^{-2} on Pt foil as the cathode.

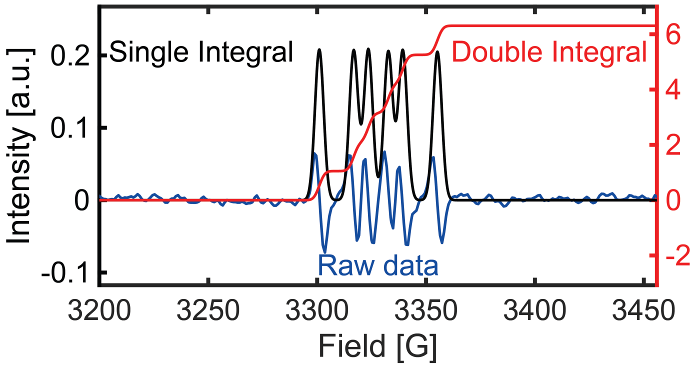

Figure S19. Qualitative assessment of free radicals in acrylonitrile electroreduction. Sample double integration of an EPR signal. The result of double integration is proportional to the number of spins in the sample.

Liquid chemical quantification

Liquid products in KIE experiments were measured using a Shimadzu gas chromatographer equipped with a mass spectrometer (GCMS-QP2010) with heptane as an internal standard. Each product was quantified using the calibration curves shown in Figure S20.

After the moles of products were determined based on the techniques outlined above, the Faradaic efficiency (FE) was calculated for each *i* species as:

$$FE_i = \frac{n_i F z_i}{Q} \quad (3)$$

where n_j is the number of moles of species *i*, *F* is Faraday's constant, z_i is the number of electrons transferred in the formation of a molecule of the species *i*, and Q is the total charge transferred. The production rate (PR) of species *i* was calculated using the following formula (where t_r is the total reaction time):

$$PR_i = \frac{n_i}{t_r} \quad (4)$$

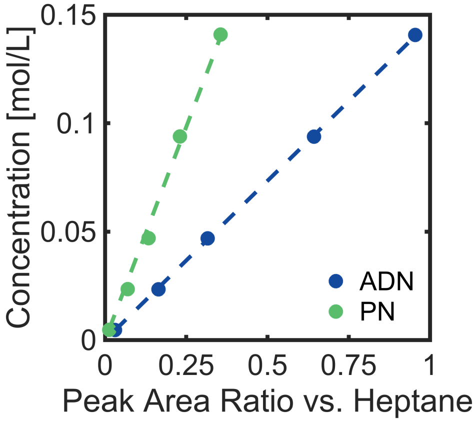

Figure S20. Gas Chromatography (GC) calibration curves for adiponitrile (ADN) and propionitrile (PN). Relating GC peak area ratio with respect to heptane (internal standard) to concentration. Each point represents an average of 2 samples.

Liquid products in free radical capture experiments were measured using a Bruker Avance III 400 NMR Spectrometer and with sodium formate (SF) as an internal standard. The moles of molecule *x* were calculated using the following formula:

$$n_x = \frac{I_x}{I_{SF}} \cdot \frac{H_{SF}}{H_x} \cdot n_{SF} \quad (5)$$

where *n*, *I*, and *H* are the moles, integral area, and number of nuclei of the compound of interest (*x*) and the calibrant (*SF*), respectively.

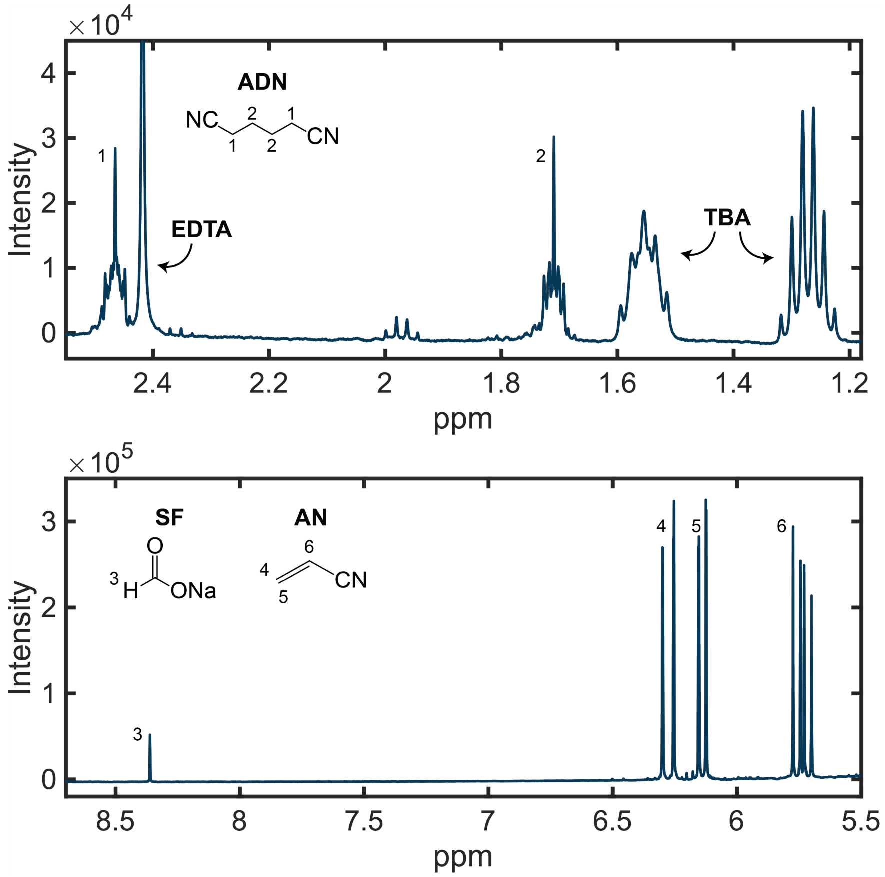

Figure S21. Sample H^1NMR spectra of relevant molecules to this study. Obtained in a 400 MHz NMR: Internal standard, sodium formate (SF); reactant, acrylonitrile (ADN); product, adiponitrile (ADN); and supporting electrolytes, ethylenediaminetetraacetic acid (EDTA) and tetrabutylammonium ion (TBA).

References

1. Atwi, R.; Bliss, M.; Makeev, M.; Rajput, N. N., MISPR: an open-source package for high-throughput multiscale molecular simulations. *Scientific Reports* **2022,** *12* (1), 15760.
2. Martínez, L.; Andrade, R.; Birgin, E. G.; Martínez, J. M., PACKMOL: a package for building initial configurations for molecular dynamics simulations. *Journal of computational chemistry* **2009,** *30* (13), 2157-2164.
3. Wang, J.; Wolf, R. M.; Caldwell, J. W.; Kollman, P. A.; Case, D. A., Development and testing of a general amber force field. *Journal of computational chemistry* **2004,** *25* (9), 1157-1174.
4. Berendsen, H.; Grigera, J.; Straatsma, T., The missing term in effective pair potentials. *Journal of Physical Chemistry* **1987,** *91* (24), 6269-6271.
5. Ryckaert, J.-P.; Ciccotti, G.; Berendsen, H. J., Numerical integration of the cartesian equations of motion of a system with constraints: molecular dynamics of n-alkanes. *Journal of computational physics* **1977,** *23* (3), 327-341.
6. Joung, I. S.; Cheatham III, T. E., Determination of alkali and halide monovalent ion parameters for use in explicitly solvated biomolecular simulations. *The journal of physical chemistry B* **2008,** *112* (30), 9020-9041.
7. Blazquez, S.; Conde, M.; Vega, C., Scaled charges for ions: An improvement but not the final word for modeling electrolytes in water. *The Journal of Chemical Physics* **2023,** *158* (5).
8. Hockney, R. W.; Eastwood, J. W., *Computer simulation using particles*. crc Press: 1988.
9. Nosé, S., A molecular dynamics method for simulations in the canonical ensemble. *Molecular physics* **1984,** *52* (2), 255-268.
10. Hoover, W. G., Canonical dynamics: Equilibrium phase-space distributions. *Physical review A* **1985,** *31* (3), 1695.
11. Hartstein, A.; Kirtley, J. R.; Tsang, J. C., Enhancement of the Infrared Absorption from Molecular Monolayers with Thin Metal Overlayers. *Physical Review Letters* **1980,** *45* (3), 201-204.
12. Hatta, A.; Suzuki, Y.; Suëtaka, W., Infrared absorption enhancement of monolayer species on thin evaporated Ag films by use of a Kretschmann configuration: Evidence for two types of enhanced surface electric fields. *Applied Physics A* **1984,** *35*, 135-140.
13. Mathison, R. FTIR Baseline Correction. https://github.com/ricardomathison/FTIR-Baseline-Correction.
14. Eaton, G. R.; Eaton, S. S.; Barr, D. P.; Weber, R. T., *Quantitative Epr*. Springer Science & Business Media: 2010.
